# Biotransformation of Selenium by Lactic Acid Bacteria: Formation of Seleno-Nanoparticles and Seleno-Amino Acids

**DOI:** 10.3389/fbioe.2020.00506

**Published:** 2020-06-12

**Authors:** Fernando Gabriel Martínez, Gustavo Moreno-Martin, Micaela Pescuma, Yolanda Madrid-Albarrán, Fernanda Mozzi

**Affiliations:** ^1^Centro de Referencia para Lactobacilos (CERELA-CONICET), Tucumán, Argentina; ^2^Departamento de Química Analítica, Facultad de Ciencias Químicas, Universidad Complutense de Madrid, Madrid, España

**Keywords:** lactic acid bacteria, selenium metabolism, probiotic, starter culture, selenocysteine, microbial cell factory, nutraceuticals

## Abstract

Selenium (Se) is an essential micronutrient for the majority of living organisms, and it has been identified as selenocysteine in the active site of several selenoproteins such as glutathione peroxidase, thioredoxin reductase, and deiodinases. Se deficiency in humans is associated with viral infections, thyroid dysfunction, different types of cancer, and aging. In several European countries as well as in Argentina, Se intake is below the recommended dietary Intake (RDI). Some lactic acid bacteria (LAB) can accumulate and bio-transform selenite (toxic) into Se-nanoparticles (SeNPs) and Se-amino acids (non-toxic). The microbial growth, Se metabolite distribution, and the glutathione reductase (involved in selenite reduction) activity of Se-enriched LAB were studied in this work. The ninety-six assayed strains, belonging to the genera *Lactococcus, Weissella, Leuconostoc, Lactobacillus, Enterococcus*, and *Fructobacillus* could grow in the presence of 5 ppm sodium selenite. From the total, eight strains could remove more than 80% of the added Se from the culture medium. These bacteria accumulated intracellularly between 1.2 and 2.5 ppm of the added Se, from which *F. tropaeoli* CRL 2034 contained the highest intracellular amount. These strains produced only the seleno-amino acid SeCys as observed by LC-ICP-MS and confirmed by LC-ESI-MS/MS. The intracellular SeCys concentrations were between 0.015 and 0.880 ppm; *Lb. brevis* CRL 2051 (0.873 ppm), *Lb. plantarum* CRL 2030 (0.867 ppm), and *F. tropaeoli* CRL 2034 (0.625 ppm) were the strains that showed the highest concentrations. Glutathione reductase activity values were higher when the strains were grown in the presence of Se except for the *F. tropaeoli* CRL 2034 strain, which showed an opposite behavior. The cellular morphology of the strains was not affected by the presence of Se in the culture medium; interestingly, all the strains were able to form spherical SeNPs as determined by transmission electron microscopy (TEM). Only two *Enterococcus* strains produced the volatile Se compounds dimethyl-diselenide identified by GC-MS. Our results show that *Lb. brevis* CRL 2051, *Lb. plantarum* CRL 2030, and *F. tropaeoli* CRL 2034 could be used for the development of nutraceuticals or as starter cultures for the bio-enrichment of fermented fruit beverages with SeCys and SeNPs.

## Introduction

Selenium (Se) is a metalloid considered a vital micronutrient in the human diet. Se replaces sulfur in cysteine and is incorporated as selenocysteine (SeCys) in selenoproteins (Mounicou et al., 2006). The main selenoenzymes are glutathione peroxidase, iodothyronine deiodinase, and thioredoxin reductase, which are involved in antioxidant defense, detoxification, and thyroid functions (Palomo-Siguero and Madrid, 2017).

Se incorporation into the body is possible through food consumption; fish and vegetables are the main sources of Se. The amount of Se intake worldwide depends on the amount present in the soil of each country and the capacity of vegetables to accumulate Se, the type of crop grown and consumed, Se speciation, soil pH, organic matter content, etc. (Rayman, 2012). Although Se has been considered a toxic element before 1973, a narrow concentration difference between Se essentiality and toxicity exists, which is dependent on its speciation (Zhang et al., 2009). Se deficiency in humans is associated with hypothyroidism, cardiovascular disease, and immune system wickedness (Pedrero et al., 2006). In nature, Se is found as selenide (Se^2−^), as elemental Se (Se^0^), and as the soluble salts selenite (${\text{SeO}}_{3}^{2-}$) and selenate (${\text{SeO}}_{4}^{2-}$), which are the most toxic forms. Some bacteria can biotransform Se salts into the seleno-amino acids selenomethionine (SeMet) and SeCys; volatile Se compounds (diethylselenide, DESe; dimethylselenide, DMSe, and dimethyldiselenide, DMDSe); and seleno-nanoparticles (SeNPs) containing mainly Se^0^ (Javed et al., 2015).

Lactic acid bacteria (LAB) inhabit a large diversity of niches such as dairy products, fermented foods, plants, soil, and the gastrointestinal and urogenital tracts, and oral cavities of humans and animals. LAB have acquired specific physiological and biochemical properties (i.e., proteolytic and lipolytic activities, tolerance to acidic conditions, etc.) to be able to survive in a wide variety of environments. These microorganisms are often suitable to be used as starter cultures for food fermentations and as probiotics for humans and animals due to their fermentative capacity, which contributes positively to food safety and the sensorial characteristics of the raw material in addition to their health-beneficial effects (Endo et al., 2018).

Some LAB can accumulate and biotransform Se salts into seleno-amino acids and SeNPs (Moreno-Martin et al., 2017; Pescuma et al., 2017); however, the metabolic pathways by which LAB bio-convert selenite remain unclear. It has been established that selenite may react with glutathione producing selenotrisulfide derivatives, which are intermediates in the conversion of inorganic Se into bioactive selenocompounds. In this reaction, reduced glutathione (GSH) is oxidized to Se diglutathione (GSSeSG) that in turn is reduced back to GSH by glutathione reductase (GR). Unstable GSSeSG is decomposed to form elemental Se [Se^0^, (Ogasawara et al., 2005)] or used as substrate for selenophosphate synthetase. The enzyme GR is a flavoprotein disulfide oxidoreductase, which catalyzes NADPH-dependent reduction of glutathione and is involved in cell defense against oxidative stress by maintaining a high intracellular GSH/GSSG (glutathione disulfide) level (Jänsch et al., 2007). On the other hand, selenocysteine lyase (SCL) is able to decompose SeCys to Se^0^ and to mobilize Se into SeCys for selenophosphate synthesis necessary for producing SeCys _t_RNA, the precursor of SeCys and selenoproteins (Lamberti et al., 2011). Both GR and SCL have been detected in some LAB (Lamberti et al., 2011; Pusztahelyi et al., 2015); however, Se metabolism has been studied mainly in dairy- or human gut-origin strains (Zhang et al., 2009; Mangiapane et al., 2014; Palomo et al., 2014; Saini et al., 2014; Deng et al., 2015; Pescuma et al., 2017; Gomez-Gomez et al., 2019).

LAB are present in vegetables and fruits in a range between 10^2^ and 10^4^ cfu/g, and although they represent a small percentage of the total microbial population represented mainly by yeasts, a broad genera and species diversity has been found in these niches (Endo et al., 2009; Di Cagno et al., 2010; Ruiz Rodriguez et al., 2019). Fruits contain high carbohydrate concentrations and low pH and protein content; environmental conditions that these bacteria have adapted to by modifying their metabolism. Within different fruit-origin LAB, fructophilic bacteria constitute a unique group, which prefers fructose over glucose consumption due to their inability to regenerate NAD^+^ through the conversion of acetaldehyde to ethanol; thus, pyruvate, fructose, or oxygen can be used as electron acceptors for NAD^+^ regeneration (Endo et al., 2018). Since selenite produces an oxidant stress response in LAB, which are mostly micro-aerophilic, the elucidation of Se metabolism in fructophilic LAB (FLAB) becomes an interesting feature (Gomez-Gomez et al., 2019). In this work, we aimed to determine the capacity of fruit-origin LAB to grow in the presence of selenite and to biotransform Se into SeNPs and Se-amino acids. Moreover, the presence of genes related to Se metabolism and the activity of GR in the presence of this metalloid in LAB were analyzed.

## Materials and Methods

### Bacterial Strains and Growth Media

Ninety-six LAB strains, belonging to the genera *Lactococcus, Weissella, Leuconostoc, Lactobacillus, Enterococcus*, and *Fructobacillus* and to twenty-one species were previously isolated from wild fruits and flowers from Northern Argentina (Ruiz Rodríguez et al., 2017; Ruiz Rodriguez et al., 2019). The cultures were stored at −20°C in MRS broth with 20% (v/v) glycerol. MRS was supplemented with 2% (w/v) fructose (MRSf) for fructophilic species (i.e., those belonging to the genus *Fructobacillus*). Before experiments, cells were transferred in fresh MRS/MRSf for LAB and FLAB, respectively, and cultured overnight (16 h) at 30°C.

### Microbial Growth in the Presence of Se

Active cultures of the studied LAB strains previously grown in MRS or MRSf were inoculated at 2% (v/v) in MRS or MRSf supplemented or not (control) with 5 ppm of Se as Na_2_SeO_3_ (Sigma-Aldrich Chemical Co., MO, USA) and incubated at 30°C for 24 h. Microbial growth was determined by following the optical density at 600 nm (OD_600_). Differences in OD_600_ at 8 and 24 h compared with initial OD were evaluated; in addition, maximum growth rates (μ_max_) were calculated as: $\mu \text{max}=\frac{\ln OD2-\ln OD1}{t2-t1}$.

To evaluate microbial colony phenotypes, strains were spread onto MRS or MRSf agar (MRS/MRSf with 2%, w/v, agar) with and without (control) 5 ppm Se. Plates were incubated at 30°C for 48 h and the color and shape of colonies were visualized.

### Biotransformation of Sodium Selenite

The capability of the assayed LAB to reduce selenite was analyzed by growing the strains in 5 ml MRS or MRSf supplemented with 20 ppm Se, as Na_2_SeO_3_ at 30°C for 24 h. Grown cultures were centrifuged at 5,600 × *g* for 5 min and the remaining ${\text{SeO}}_{3}^{2-}$ concentration in the supernatants was determined spectrophotometrically by the modified method of Brown and Watkinson (Kessi et al., 1999) using a microplate assay. Briefly, 160 μl of 0.5 M HCl, 80 μl of 0.1 M EDTA, 40 μl of 0.1 M NaF, and 40 μl of 0.1 M dipotassium oxalate were mixed in a 1.5-ml microtube. Then, 500 μl of each culture supernatant and 200 μl of 0.1% 2,3-diaminonaphthalene in 0.1 M HCl were added. After mixing, the microtubes were incubated at 40°C for 40 min and then cooled in ice bath. A volume of 500 μl of cyclohexane was utilized for extracting the Se-2,3-diaminonaphthalene complex by shaking the microtubes placed horizontally in an orbital shaker for 10 min at 850 × g. Then, samples were centrifuged at 3000 × g for 10 min. Aliquots of 150 μl of the organic phase were placed in a 96-well microplate and the absorbance at 377 nm was determined with a Versamax Microplate reader (Molecular Devices, CA, USA). The whole reaction procedure was done in the dark. Calibration curves were obtained by adding 0, 2.5, 5.0, 10, 20, and 30 ppm Se in MRS broth. All measurements were done in triplicate.

### Intracellular Se Accumulation as Determined by Inductively Coupled Plasma Mass Spectrometry (ICP-MS)

Cells were grown in MRS or MRSf at 30°C for 24 h in the absence or presence of 5 ppm Se and then centrifuged at 4,600 × g for 5 min. For this purpose, supernatants and bacterial cell pellets (0.1 g obtained from 10 ml culture) were submitted to acid digestion in a 1000-W microwave oven (MSP microwave oven, CEM, Matthews, NC, USA) using closed vessels containing 1 ml of concentrated HNO_3_ and 0.5 ml of 30% (v/v) H_2_O_2_ (Pescuma et al., 2017). The resulting solutions were cooled, diluted to a 25-ml final volume with MilliQ water and analyzed for the Se concentration with an Agilent 7700-collision/reaction cell ICP-MS (Agilent Technologies, Santa Clara, CA, USA). Hydrogen gas was employed as collision gas for Se determination. Optimal operating conditions are included in Table 1. A control group of each bacterial species unexposed to Se was performed in parallel. Three independent replicates were made; the reaction mixture without the addition of cell pellets was used as a blank and considered in the final results.

**Table 1:** Operating conditions for LC-ICP-MS.

**Operating Conditions**	
**ICP-MS parameters for Se determination**	
RF power (W)	1,550
Plasma gas flow rate (L/min)	15.0
Ar auxiliary flow rate (L/min)	0.30
Carrier gas flow rate (L/min)	0.75
Nebulizer	Slurry
Spray chamber	Scott
Acquisition mode	Continuous
Isotopes monitored	^76^Se, ^77^Se, ^78^Se, ^80^Se
Replicates	3
Reaction gas	H_2_
Reaction gas flow rate (ml H_2_/min)	6
**AEX chromatographic parameters**	
Column	Hamilton PRP-X100 (150 mm × 4.6 mm, 10 μm)
Mobile phases	Ammonium citrate 10 mM, 2% MeOH (pH 5.0)
Mode	Isocratic
Flow rate (ml/min)	1
Injection volume (μl)	100
**RP chromatographic parameters**	
Column	Kinetex EVO C18 (150 mm × 3.0 mm, 5 μm)
Mobile phases	0.1% Formic acid, 0.5% MeOH
Mode	Isocratic
Flow rate (ml/min)	0.5
Injection volume (μl)	20

### Selenium Species Determination by Liquid Chromatography-ICP-MS (LC-ICP-MS)

Selenium species were determined in enzymatically hydrolyzed bacterial cell pellets previously grown in 5 ppm Se MRS/MRSf by LC-ICP-MS (Pescuma et al., 2017). Cell pellets (0.1 g obtained from 10 ml culture) were mixed with 600 μl of 10 mg/ml lysozyme (Sigma-Aldrich Chemical Co.) in Tris–HCl buffer, pH 7.0, and incubated at 37°C for 3 h. Then, cell suspensions were sonicated with an ultrasonic probe (Sonoplus ultrasonic homogenizer, Bandenlin, Berlin, Germany) using 6 cycles of 50 s at 60% of ultrasound amplitude. Afterwards, 400 μl of 2 mg/ml protease type XIV (Sigma-Aldrich Chemical Co.) in Tris–HCl buffer was added and incubated overnight at 37°C. Finally, mixtures were centrifuged at 7,000 × g for 15 min and the supernatants were collected, filtered through 0.22 μm nylon membrane filters, and analyzed by LC-ICP-MS by using an anion exchange column (Hamilton PRP-X100, 250 × 4.1 mm, 10 μm) following the experimental conditions given in Table 1. The following five Se species: the seleno-amino acids, selenomethionine (SeMet), selenomethylcysteine (SeMetCys), and selenocystine (SeCys_2_), as well as two inorganic salts, sodium selenite (Na_2_SeO_3_) and sodium selenate (Na_2_SeO_4_), were analyzed in this study. Identification of Se species was carried out by matching retention times and by spiking experiments. A calibration curve was performed for each standard species. Three independent replicates were made for each sample; the enzymatic mixture without the addition of bacterial cell pellets was used as a blank and considered in the final results.

### Confirmation of the SeCys Species by LC-Tandem Mass Spectrometry (LC-ESI-MS/MS)

Carbamidomethylation was used for preserving SeCys integrity according to Palomo-Siguero et al. (2016) with slight modifications. The procedure was applied before enzymatic digestion. *F. tropaeoli* CRL 2034 was grown in MRSf with 5 ppm Se at 30°C for 24 h and then the culture was centrifuged at 4600 × g for 5 min. Pellets were washed twice with MilliQ water and suspended in 1 ml of 0.1 M Tris–HCl buffer, pH 7.5. A reduction of S–S, Se–S, and Se–Se bridges of the proteins was performed by incubating at 37°C with 60 μl of 0.2 M dithiothreitol (DTT) in the dark followed by alkylation with 100 μl of 0.5 M iodoacetamide in the same conditions for 1.5 h in each step. To remove the excess of iodoacetamide, 400 μl of DTT was added and shaken at 200 × g for 1 h. After carbamidomethylation, samples were submitted to enzymatic digestion as described above. The extracts were centrifuged at 7,000 × *g* for 15 min; supernatants were filtered using 0.22-μm nylon membrane filters and stored at −80°C until analysis. To evaluate the correct formation of carbamidomethyl-SeCys (CAM-SeCys), Se species were analyzed by LC-ICP-MS using a Phenomenex Kinetex EVO C18 column following the experimental conditions given in Table 1. Confirmation of CAM-SeCys was done with LC-ESI-MS/MS using the same reversed-phase chromatographic column and conditions as for LC-ICP-MS. Analyses were carried out with a Shimadzu LC-MS-8030 triple quadrupole system (Shimadzu Scientific Instrument, Columbia, MD, USA) supplied with a Nexera LC-30AD solvent delivery unit, a Nexera SIL 30AC autosampler with a temperature-controlled tray, and a CTO- 20AC column oven. The equipment was operated in positive electrospray ionization (ESI) mode. Nitrogen was used as nebulizing (1.5 L·min^−1^) and drying (15.0 L·min^−1^) gas. Collision-induced dissociation was done using argon as collision gas at a pressure of 230 kPa in the collision cell with a collision energy voltage of 25 eV. Ionization voltage for ESI was set at 4.5 kV; the interface current was fixed at 4.4 μA, and the detector voltage was fixed at 2.10 kV. Due to lack of standard for CAM-SeCys species, a bibliographical search was made with the aim of selecting the most common transitions. As precursor ion [M + H]^+^, was selected and the detection was carried out in multiple reaction monitoring (MRM) mode, with a dwell time of 100 ms, by monitoring three or four selective transitions for each species.

### Extraction and Determination of Volatile Se Compounds by HS-SPME-GC-MS

The volatile Se compound production by eight selected strains was evaluated following the method described by Moreno-Martin et al. (2019). Cells were cultured in 50 ml of MRS or MRSf supplemented with 5 ppm Se at 30°C for 24 h in 250-ml Erlenmeyer flasks sealed with a silicone septum. The analytical procedure for volatile Se compound determination consisted of two steps: extraction by headspace solid-phase microextraction (HS-SPME) followed by separation and analysis by gas chromatography coupled to mass spectrometry (GC-MS). In the extraction step, a 75-μm fiber coated with Carboxen/PDMS (Supelco, Bellefonte, PA, USA) was inserted into each Erlenmeyer flask through the septum and placed statically in an incubator at 30°C. Analyte extraction was carried out in the headspace for 26 min. Immediately after the extraction, separation and analysis of the resulting extracts were performed in an Agilent 7890A/5975C GC-MS (Agilent Technologies S.A., Madrid, Spain) instrument. High-purity helium (>99.999%) was used as carrier gas at a flow rate of 0.5 ml/min. A 0.75 mm ID SPME Inlet Liner (Supelco, Bellefonte, PA, USA) was used with splitless mode injections. The inlet temperature was set at 300°C and desorption was done at a purge flow of 60 ml/min for 0.25 min, and the septum purge flow was 3 ml/min. A polydimethylsiloxane (95%) cross-linked ZB5MS (Zebron, Phenomenex, Madrid, Spain) capillary column was used (30 m × 0.25 mm ID, 0.25 μm d_f_); the oven temperature program was as follows: from 40°C (3 min) to 180°C (5 min) at 10°C/min and from 180°C to 200°C (1 min) at 30°C/min. The mass spectrometer conditions were as follow: 150°C of quadrupole temperature, 280°C of transfer line temperature, and 230°C of ion source temperature. SCAN mode with an acquisition range of 25–300 m/z was selected for the identification of the Se volatile compounds based on the retention times as well as the fragmentations of standard solutions. Three volatile Se compounds were analyzed: dimethyl selenide (DMSe), dimethyl diselenide (DMDSe), and diethyl selenide (DESe) (Sigma-Aldrich Chemical Co.). These compounds were dissolved in H_2_O:MeOH (50:50, v/v) with pure water (18 MΩ/cm) from a Milli-Q system (Millipore, Bedford, MA, USA) and MeOH (HPLC grade, Scharlab, Barcelona, Spain). The following fragments, referred to the mass to charge ratio (m/z), were selected in SIM mode for the identification of volatile Se compounds: m/z 80, 95 and 110 for DMSe, m/z 80, 95, 110 and 138 for DESe, and m/z 95, 160, 175, and 190 for DMDSe. Data were acquired using MSD ChemStation software and analyzed in an MSD ChemStation Data Analysis Application (Agilent Technologies S.A., Madrid, Spain). Identification of Se volatile compounds was done based on the 2011 NIST library and supported by corrected retention times and fragmentation standard (Moreno-Martin et al., 2019).

### GR Activity

Strains were grown in MRS/MRSf in the absence or presence of 5 ppm Se at 30°C for 24 h; cells were harvested by centrifugation and washed with 0.1 M sodium phosphate buffer (pH 7.5) with 1 mM EDTA. Then, microbial cells were mixed with the same buffer and glass beads (150–212 μm diameter; Sigma-Aldrich Chemical Co.) in a 1:2:1 ratio (cells:buffer:glass beads, w/v/w). Cells were lysed by mechanical beating after loading the vials in a Mini-Bead Beater-8 cellular disruptor (Biospec Products Inc., Bartlesville, OK, USA) with intermittent cooling on ice-bath (6 cycles of 1.5 min). The cell debris were removed by centrifugation and lysates were used for GR activity assay (Pophaly et al., 2017). The assay depends upon the transformation of GSSG to GSH with concomitant oxidation of 5,5′-Dithiobis(2-nitrobenzoic acid) (DTNB, Sigma-Aldrich Chemical Co.) to a colored compound. Briefly, the reaction mixture contained 150 μl of 0.6 mg/ml DTNB, 10 μl of 10 mg/ml NADPH (Sigma-Aldrich Chemical Co.), and 20 μl of cell-free extract. The reaction was initiated by adding 10 μl of 1 mg/ml GSSG (Sigma-Aldrich Chemical Co.). All solutions were made in 0.1 M sodium phosphate buffer (pH 7.5) with 1 mM EDTA. Absorbance was monitored at 405 nm over a period of 4 min in a 15-s time interval in a microplate reader (Versamax, Molecular Devices, USA).

### Genes Involved in Se Metabolism

PCR assay was used to investigate the presence of genes involved in Se metabolism. Genes coding for GR (*GshR*/*gor*) and selenocysteine lyase (*Scl*) were sought in the genome of eight strains in this work. Genomic DNA was extracted from selected strains according to Ruiz Rodriguez et al. (2019). Five milliliters of stationary phase cultures were centrifuged at 7000 × *g* for 5 min. Cells were washed with 500 μl of TE buffer [10 mM Tris–HCl (pH 7.5), 10 mM EDTA] and resuspended in 1.7 ml of lysozyme (15 mg/ml) and dissolved in SET buffer [20 mM Tris–HCl (pH 7.5), 25 mM EDTA, 75 mM NaCl]. After holding at 37°C for 2 h, 170 μl of 10% (w/v) sodium dodecyl sulfate (SDS) and 50 μl of proteinase K (15 mg/ml) were added, and the mixture was incubated at 55°C for 2 h. Then, 700 μl of 5 M NaCl and 2.7 ml of chloroform:isoamyl alcohol (24:1) were added to the mixture, maintaining it at room temperature for 30 min. After centrifugation at 11,200 × *g* for 10 min, the aqueous phase was transferred to another tube, and the DNA was precipitated with 2.5 ml of isopropanol. The precipitate was washed with 1 ml of 70% (v/v) ethanol and centrifuged for 10 min at 11,200 × *g*. DNA was dried by evaporating the alcohol and then resuspended in 30 μl of MilliQ water. DNA concentration and purity were spectrophotometrically determined by measuring the OD at 260 and 280 nm and determining the OD_260_/OD_280_ ratio (Brown, 1995).

The PCR assay mixture (25 μl) consisted of 5 μl of 5 × Green GoTaq® Reaction Buffer (Promega, Madison, WI, USA), 1 μl of a mixture of dNTPs (dATP, dTTP, dCTP, and dGTP, 5 mM), 0.2 μl of 5 U/μl GoTaq® DNA Polymerase (Promega), 2.5 μl of 10 μM each primer, 9.8 μl of nuclease-free water, and 4 μl of the purified chromosomal DNA (50 ng/μl) as template. Primers were designed by searching each gene sequence for the corresponding bacterial species in the National Center for Biotechnology Information (NCBI) nucleotide database; the sequences found were aligned and compared, and the conserved region of the gene sequences were used to design the primers. PCR amplifications were performed with a My Cycler^TM^ thermal cycler (Bio-Rad Laboratories, Inc., Hercules, CA, USA) using the following program: 94°C for 4 min, 30 cycles of 94°C for 30 s, accurate annealing temperature for 30 s, and 72°C for 45 s, and a final extension step at 72°C for 5 min. Primers and their corresponding melting temperatures used in this work are listed in Table 2. PCR products were electrophoresed in a 0.8% (w/v) agarose gel at 80 V for 45 min in 1 × TAE buffer and then, were visualized after staining with GelRed^TM^ Nucleic Acid Gel Stain (Biotium, Hayward, CA, USA) under a UV trans-illuminator and digital image documentation using a CCD camera (ChemiDoc^TM^ XRS+ System, Bio-Rad Laboratories, Inc., CA, USA), and images were recorded using Image Lab^TM^ Software. The size of DNA fragments was estimated using a standard 1-kb DNA ladder (1Kb Plus DNA Ladder, Invitrogen^TM^, Carlsbad, CA, USA). Amplicons were purified by polyethylene glycol precipitation (protocol available at http://gator.biol.sc.edu/; http://labs.mcdb.lsa.umich.edu/labs/olsen/files/PCR.pdf). Nucleotide sequences of purified PCR products were determined at the CERELA sequencing facility with an ABI 3130 DNA sequencer (Applied Biosystems, Foster, CA, USA).

**Table 2:** Primer sequences for genes coding for glutathione reductase and selenocysteine lyase genes.

	**Name**	**Sequence (**5^′^ → 3^′^)****	**Annealing temperature**	**Amplicon length (bp)**
Glutathione reductase	GRL-FW	GGGAGGAACTTGTCCAAACTAT	50	663
	GRL-RV	CCTACTCCCGCAATCACTAAAT		
	GRW-FW	CAACAAGCCAGCCACATAAC	50	673
	GRW-RV	CTTGGACGATGCGGGTATTA		
	GRW2-FW	CTCAATGGCTGGCAACAAAG	50	819
	GRW2-RV	ACGATACCAGGTGCAGATTTAG		
	GRE-FW	GCGAGGCGTAGAAGTCAAA	50	575
	GRE-RV	ATGCTTGTGTCTGGATCGTATAG		
	GRP-FW	AATGACGGCTGATGGGATTAC	50	324
	GRP-RV	GTCAACTCACCGACCAGATAAC		
	GRF-FW	CCAAGAACATGGCCCGTAAA	50	776
	GRF-RV	TGTGGTACCGATAGCTGGATAG		
Selenocysteine lyase	LyaseL-FW	ACAAGACAACAGGAGCAGTATC	50	486
	LyaseL-RV	ACGGTGTCGCAAAGGTATTAT		
	LyaseW-FW	GGATAGAGACACCGTCAAACC	50	453
	LyaseW-RV	CCTCCGCTAAGTAGAACGAAAT		
	LyaseE-FW	CAGCTATCATGAGGCCCTAATAC	50	602
	LyaseE-RV	CTTGGTCAGCCAAAGCAATATG		
	LyaseB-FW	CTTGGCAAACCCACGATAGA	40	678
	LyaseB-RV	GTACAACCCTTGGCTGAAATTG		
	LyaseF-FW	TGAACACCCGCTAGGTTAAAG	50	454
	LyaseF-RV	GGGCAACGGTTATTGTTGATG		

*GR, glutathione reductase; Lyase, selenocysteine lyase; -FW, forward; -RV, reverse; L, Lactococcus; W, Weissella; E, Enterococcus; P, Lb. plantarum; F, Fructobacillus; B, Lb. brevis*.

### Detection of Se Nanoparticles by Transmission Electron Microscopy

The presence, composition, and size of Se nanoparticles (SeNPs) were assessed by TEM. The studied strains were grown in MRS or MRSf supplemented with 5 ppm Se at 30°C for 24 h. Samples were prepared by placing a drop of culture onto a 300-mesh lacey carbon copper TEM grids. The film on the TEM grids was allowed to dry for 5 min at room temperature before analysis. Transmission electron micrographs were recorded using a high-resolution transmission electron microscope (JEM-2100, JEOL USA, CA, USA) equipped with an X-ray energy dispersive spectroscopy (XEDS) microanalysis composition system (Oxford Inc.). Analysis of SeNP composition was carried out by XEDS microanalysis (Dhanjal and Cameotra, 2010). The diameter of SeNPs (*n* = 100) was measured from the obtained images by using the free image-processing software, ImageJ (version 1.52a, Java 1.8.0_112, Wayne Rasband, National Institutes of Health, USA; website: https://imagej.nih.gov/ij/).

### Visualization of LAB Cells and SeNPs by Scanning Electron Microscopy

The cell morphology of *Lactobacillus brevis* CRL 2051, *Lb. plantarum* CRL 2030, and *Fructobacillus tropaeoli* CRL 2034 (which accumulated the highest amounts of SeCys) and the formation of SeNPs by these strains were visualized by scanning electron microscopy (SEM) with a Zeiss Supra 55VP instrument (Oberkochen, Germany) in the CISME-CCT-CONICET electron microscopy facility, San Miguel de Tucumán, Argentina. For this purpose, LAB strains were grown in MRS or MRSf with 5 ppm Se at 30°C for 24 h, centrifuged, and washed three times with saline solution. Fixation was done by suspending the cells with 4% (v/v) glutaraldehyde solution, and then suspensions were layered onto solid agar-coated SEM coverslips. Fixed cells were dehydrated through a series of alcohol dehydration steps (30, 50, 70, 90, and 100%). Finally, the samples were coated with gold and visualized under SEM.

### Statistics

Assays were carried out in triplicate and results were expressed as the mean ± SD. TEM and SEM images were selected from independent assays for each strain. One-way analysis of variance (ANOVA) and Tukey's post comparison test using MINITAB 16 Statistical Software (Minitab, State College, PA, USA) was applied for statistical analysis.

## Results

### Microbial Growth in the Presence of Se

We studied the growth behavior of ninety-six fruit- and flower-origin LAB strains belonging to 6 genera and 21 different species in MRS or MRSf (for FLAB) in the absence or the presence of 5 ppm Se. All strains were able to grow in the presence of Se at the evaluated concentration although different growth behaviors were noticed depending on the assayed strains (Table S1). The presence of Se did not significantly affect the growth of 41 strains, which included those belonging to the species *Weissella minor* (9), *Leuc. pseudomesenteroides* (18 out of 28), and *F. tropaeoli* (6). In general, all fructobacilli strains, including the four studied species, showed the highest cell growth rates in the presence of Se, while the highest cell growth values after 24 h corresponded to both the fructobacilli and the majority of the *Leuc. pseudomesenteroides* strains. From the total, the growth parameters of only 10 strains were significantly affected in the presence of Se, among which the four *Ec. hirae* strains were included. The most negatively affected strains by Se were *Lc. lactis* subsp. *lactis* CRL 2009, *Leuc. mesenteroides* CRL 2059, *Leuc. pseudomesenteroides* CRL 1997, and *Lb. rhamnosus* CRL 2031, which showed a deleterious effect (more than 50% alteration) in at least one of the growth parameters evaluated.

LAB colonies showed their typical translucent white color in MRS and MRSf agar, while in the presence of 5 ppm Se, all the evaluated strains showed a reddish color indicating their ability to reduce the colorless Na_2_SeO_3_ salt to elementary Se (Se^0^, orange-reddish); examples of reddish colonies are shown in Figure S1. Colonies changed from white to red color after 24 h of incubation, increasing the intensity of the reddish color over time. After 48 h of incubation, no color change was further observed.

### Biotransformation of Sodium Selenite

To evaluate the ability of the assayed 96 LAB strains to biotransform and intracellularly accumulate Se, the remaining selenite concentration in 24-h culture supernatants was determined spectrophotometrically. The ability of the strains to remove Se from the culture medium was variable (between 8 and 100% of the added selenite) and strain- and species-dependent (Table 3). The species *Lc. lactis, W. cibaria, Ec. casseliflavus*, and *F. tropaeoli* removed the greater Se amounts (>70%) from the culture medium compared to the other species within each genus. Interestingly, while several strains of *Ec. casseliflavus* showed 100% Se removal, the strains of *Ec. durans* and *Ec. faecium* showed an opposite behavior by scarcely removing Se in amounts lower than 27%. Regarding the *Leuconostoc* genus, all species removed low to moderate amounts (between 14 and 71%). In general, the *Lactobacillus* strains removed low amounts (<45%) of selenite from the culture medium, except for *Lb. brevis* CRL 2051 and *Lb. plantarum* CRL 2030, which removed both more than 95% of the added Se.

**Table 3:** Percentage of selenite removal from the culture medium by the studied LAB strains.

**Bacterial strains**		**Percentage removal of selenite by each strain**				
*Lactococcus*	*lactis subsp. lactis*	76 ± 11	93 ± 1	104 ± 5		
	*lactis subsp. cremoris*	81 ± 5				
	*lactis*	34 ± 1				
*Weissella*	*cibaria*	85 ± 4	84 ± 8	74 ± 4	84 ± 3	
	*fabalis*	57 ± 1				
	*minor*	31 ± 4	24 ± 3	15 ± 2	24 ± 3	28 ± 4
		23 ± 2	49 ± 3	22 ± 3	30 ± 4	
*Leuconostoc*	*mesenteroides subsp. mesenteroides*	39 ± 2	56 ± 6	28 ± 4	46 ± 2	43 ± 2
	*mesenteroides*	37 ± 4	42 ± 3	28 ± 4	28 ± 4	30 ± 2
	*pseudomesenteroides*	42 ± 6	43 ± 2	40 ± 6	32 ± 4	39 ± 6
		46 ± 3	54 ± 5	57 ± 8	28 ± 2	38 ± 1
		46 ± 3	19 ± 2	50 ± 4	42 ± 3	52 ± 2
		45 ± 3	44 ± 3	49 ± 2	14 ± 2	58 ± 2
		52 ± 2	44 ± 4	64 ± 2	71 ± 11	43 ± 2
		60 ± 8	49 ± 3	68 ± 10		
	*citreum*	62 ± 3	59 ± 5			
*Lactobacillus*	*brevis*	37 ± 5	95 ± 3	33 ± 5	46 ± 1	43 ± 3
		40 ± 3	43 ± 2			
	*plantarum*	98 ± 6				
	*rhamnosus*	24 ± 3	26 ± 4			
*Enterococcus*	*casseliflavus*	102 ± 4	101 ± 1	101 ± 1	100 ± 1	104 ± 2
		58 ± 2	84 ± 3	101 ± 4		
	*faecalis*	86 ± 13				
	*hirae*	62 ± 4	72 ± 4	47 ± 4	47 ± 7	
	*mundtii*	39 ± 2				
	*faecium*	22 ± 3	27 ± 4	26 ± 3		
	*durans*	8 ± 1				
*Fructobacillus*	*durionis*	41 ± 3				
	*fructosus*	52 ± 6				
	*pseudoficulneus*	57 ± 3				
	*tropaeoli*	75 ± 6	71 ± 3	79 ± 4	63 ± 2	80 ± 4
		3 ± 2				

*Each value corresponds to a different strain. Results are mean values with standard deviations. Color scale represents different values, red meaning higher and green lower ones*.

### Intracellular Se Accumulation as Determined by ICP-MS

**Figure 1:**
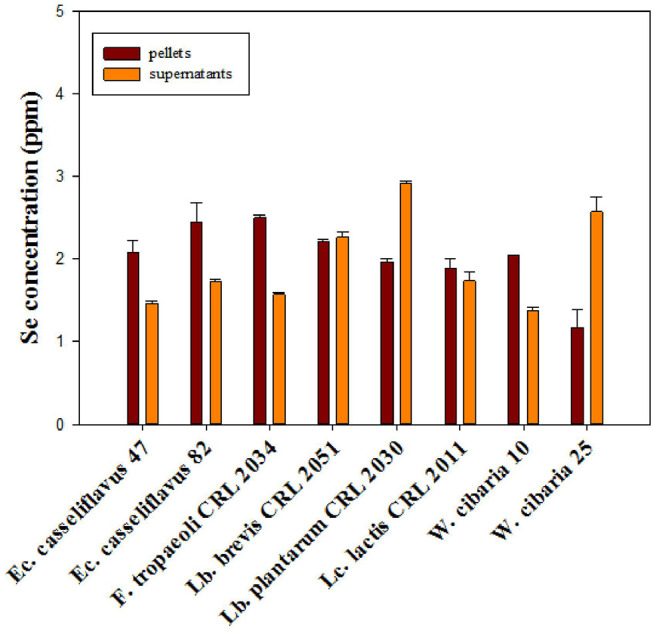
Total Se content in cell pellets and supernatants of selected LAB strains grown in MRS and MRSf with sodium selenite at 30°C for 24 h, as revealed by ICP-MS.

From the total assayed LAB, eight strains, namely, *Lc. lactis* CRL 2011, *W. cibaria* 10 and 25, *Ec. casseliflavus* 47 and 82, *Lb. brevis* CRL 2051, *Lb. plantarum* CRL 2030, and *F. tropaeoli* CRL 2034, were selected for further experiments due to their capacity to grow in the presence of Se and to reduce more than 80% of the added selenite in the culture medium. The strains grew similarly (8.5–9.1 log CFU/ml) and could intracellularly accumulate between 1.2 and 2.5 ppm Se (red bars, Figure 1) after 24-h incubation as revealed by ICP-MS. Considering the cell pellet weights, the assayed strains accumulated between 126 and 580 μg Se/g cell; the highest amount corresponded to *F. tropaeoli* CRL 2034 while the lowest corresponded to *Lb. brevis* CRL 2051. The remaining Se content in the cell-free supernatants was greater in samples of *Lb. plantarum* CRL 2030 and *W. cibaria* 25. Whereas, the two enterococci samples showed similar values, those of the two *W. cibaria* strains showed a strain-dependent behavior.

### Determination of Se Species in Cell Pellets of LAB Strains by LC-ICP-MS

**Figure 2:**
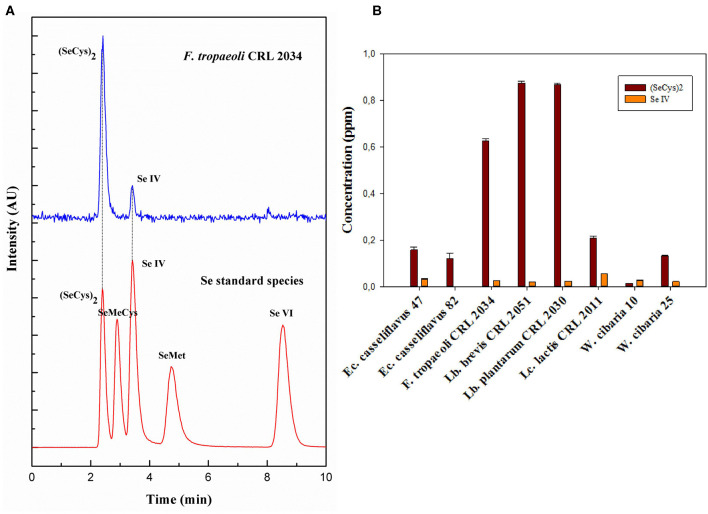
Se species in enzymatically hydrolyzed LAB cell pellets. **(A)** Anion exchange ICP-MS chromatograms of standard Se species, and Se species formed by *F. tropaeoli* CRL 2034 (as example) are represented in red and blue, respectively. **(B)** Se species concentration for the assayed LAB. AU, arbitrary units.

Selenium species in the enzymatic extracts were determined by LC-ICP-MS using a PRP-X100 anion exchange column. Identification of Se species was done by comparing the retention times and spiking experiments with Se standard species (Figure 2A); Se species concentration was calculated with the obtained calibration curve performed for each standard species. For all the assayed strains, SeCys_2_ was detected at 2.4 min as the main Se species with concentration values between 0.01 and 0.87 ppm (Figure 2B). Traces of selenite (Se IV) from the culture medium were also detected at 3.3 min in the majority of the cell pellets, although in very low concentrations (<0.05 ppm). Only for the strain *Ec. casseliflavus* 82, selenite was not detected. *Lb. brevis* CRL 2051, *Lb. plantarum* CRL 2030, and *F. tropaeoli* CRL 2034 accumulated the highest intracellular SeCys_2_ values (39, 44, and 25%, respectively), highlighting the ability of these strains to incorporate Se as SeCys_2_. Similar SeCys_2_ concentrations were observed for the enterococci while different values were noticed for the *Weissella* strains.

### Confirmation of the Presence of SeCys Species as Revealed by LC-ESI-MS/MS

**Figure 3:**
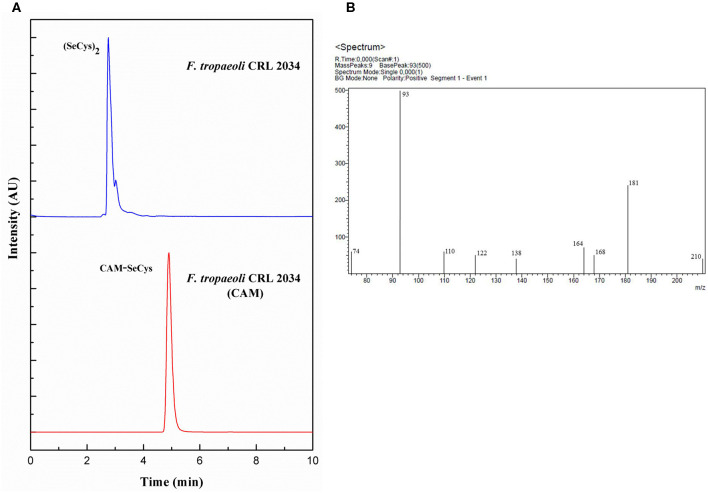
Detection of CAM-SeCys in the cell pellet of *F. tropaeoli* CRL 2034: **(A)** Chromatograms obtained from reversed-phase column coupled to ICP-MS, *F. tropaeoli* CRL 2034 with and without applying the carbamidomethylation procedure are represented in red and blue, respectively; **(B)** Fragmentation of CAM-SeCys by LC-ESI-MS/MS. AU, arbitrary units; CAM, carbamidomethylated.

To corroborate that the detected Se species SeCys_2_ by LC-ICP-MS effectively corresponded to SeCys, carbamidomethylation of this compound (CAM-SeCys) was done using the cell pellet of the strain *F. tropaeoli* CRL 2034. Comparison of CAM-SeCys retention time to that of the carbamidomethylated standard was carried out using a reversed-phase C_18_ column coupled to ICP-MS since its mobile phase is compatible with the LC-ESI-MS/MS analysis used for identifying CAM-SeCys. Only one intense peak at 4.9 min corresponding to CAM-SeCys (Figure 3A) was observed. Further, CAM-SeCys was confirmed by comparing the fragmentation ions of the sample obtained by LC-MS/MS (Figure 3B) with those theoretical ion fragments reported for CAM-SeCys by Dernovics and Lobinski (2008). All theoretical peaks corresponding to CAM-SeCys were detected in the sample and listed in Table 4; confirming that the peak obtained at 4.9 min corresponded to the presence of CAM-SeCys (and consequently confirming SeCys as the major Se species found in Se-treated bacteria).

**Table 4:** Description of the proposed fragment ions formed during the fragmentation of cabamidomethyl-SeCys, *m/z* 226.9 ([M + H]^+^).

**No**.	**Elemental composition**	**Theoretical mass/Th^**a**^**	**Measured mass/Th^**a**^**	**Notes on losses and fragments**
1	C_5_H_8_NO_3_Se^+^	209.9664	209.9	NH_3_
2	C_4_H_9_N_2_OSe^+^	180.9875	180.9	Formic acid
3	C_3_H_6_NO_2_Se^+^	167.9564	167.9	Alkylating group
4	C_4_H_6_NOSe^+^	163.9609	163.9	Formic acid and NH_3_
5	C_2_H_4_NOSe^+^	137.9453	137.9	-
6	C_2_H_4_NSe^+^	121.9503	121.9	Formic acid and alkylating group
7	CH_4_NSe^+^	109.9503	109.9	NH_3_ addition to fragment No. 8
8	CHSe^+^	92.9238	92.9	-
9	C_2_H_4_${\text{NO}}_{2}^{+}$	74.0237	74.0	-

a *Th, Thomson, where 1 Th, 1 m/z. All the indicated losses are related to the intact Se species*.

### Production of Volatile Se Compounds by LAB Strains

**Figure 4:**
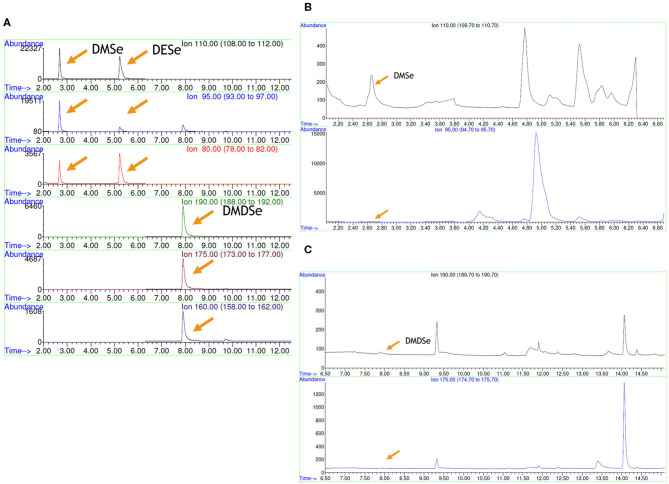
Volatile Se compounds determined by HS-SPME-GC-MS. **(A)** Standard solutions, **(B)** strain *Ec. casseliflavus* 82, and **(C)** *Ec. casseliflavus* 47. DMSe, dimethyl selenide; DESe, diethyl selenide; DMDSe, dimethyl diselenide.

Identification of volatile Se species produced by selected LAB strains grown in presence of selenite was determined by HS-SPME-GC-MS. Only the two enterococci strains produced volatile Se compounds; DMDSe and DMSe were formed by *Ec. casseliflavus* 47 and 82, respectively, albeit at very scarce amounts, which were under the limit of quantification (LOQ) of the technique. These volatile Se compounds were identified based on retention times and m/z ratios by comparing them with their respective standards. It should be noted that, for these compounds, two out of three fragments were found. The production of DMSe (2.64 min) could be identified by the increase in the signal in m/z 110 and 95 fragment ions, while no signal was found at this time for the ionic fragment m/z 80, whereas for DMDSe (7.90 min), m/z 160 was not found in the chromatograms while a slight signal was detected for fragments m/z 190 and 175 (Figure 4). The presence of the species DESe was not detected in anyone of the assayed strains.

### GR Activity

**Figure 5:**
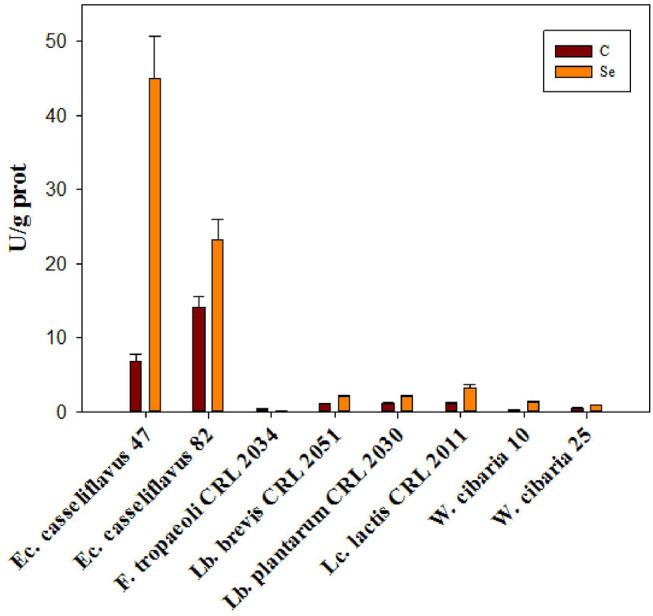
Glutathione reductase activity of eight selected LAB strains grown in MRS and MRSf with and without 5 ppm Se at 30°C for 24 h.

GR activity was determined in the eight selected LAB strains; the GR activity values were higher (between 7 and 14 U/g protein) in the absence of Se for the *Enterococcus* strains. In general, a two-fold increase in the presence of Se was detected except for *Ec. casseliflavus* 47, which showed a six-fold increase (from 7 to 45 U/g protein) compared to non-selenized cells (Figure 5). An opposite behavior was observed for *F. tropaeoli* CRL 2034 as a three-fold higher GR activity was detected in the absence of Se than when cells were selenized.

### Detection of Genes Involved in Se Metabolism

**Figure 6:**
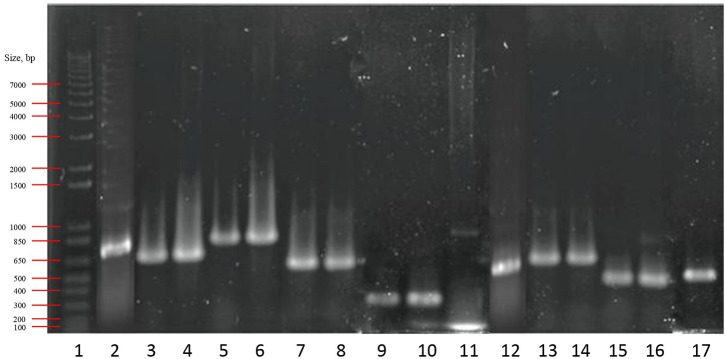
PCR products of the glutathione reductase and selenocysteine lyase genes. Lanes: 1, molecular weight marker (1 Kb Plus DNA Ladder; Invitrogen^TM^, CA, USA); 2, 3, 4, 5, 6, 7, 8, 9, 10, and 11, glutathione reductase; 12, 13, 14, 15, 16, and 17, selenocysteine lyase. Lanes 2 and 12 correspond to *Lc lactis* CRL 2011; 3, 5, and 15, *W. cibaria* 10; 4, 6, and 16 *W. cibaria* 25; 7 and 13, *Ec. casseliflavus* 47; 8 and 14, *Ec. casseliflavus* 82; 9, *Lb. brevis* CRL 2051; 10, *Lb. plantarum* CRL 2030; while lanes 11 and 17 correspond to *F. tropaeoli* CRL 2034.

In this work, the presence of two important genes involved in Se metabolism was studied by PCR reactions, i.e., genes coding for GR, *GshR*/*gor* and for selenocysteine lyase, *Scl*. Primers used are listed in Table 2. Amplification fragments for *GshR*/*gor* were detected in all studied strains (Figure 6). Two different *GshR*/*gor* bands of 673 and 819 bp were detected for the *W. cibaria* species, which were amplified by using different primers (GRW and GRW2). The *Scl* genes coding for the enzyme SCL were detected in all strains (amplicon length from 453 to 678 bp), except for the two studied *Lactobacillus* (Figure 6), which were not amplified with the primers used (LyaseB). In all cases, the identity of the obtained PCR amplicons was further confirmed by sequence analysis. The size of DNA fragments was estimated using a standard 1-kb DNA ladder and was accurate with the expected length of the amplicons (Table 2).

### Detection of SeNPs by TEM

**Figure 7:**
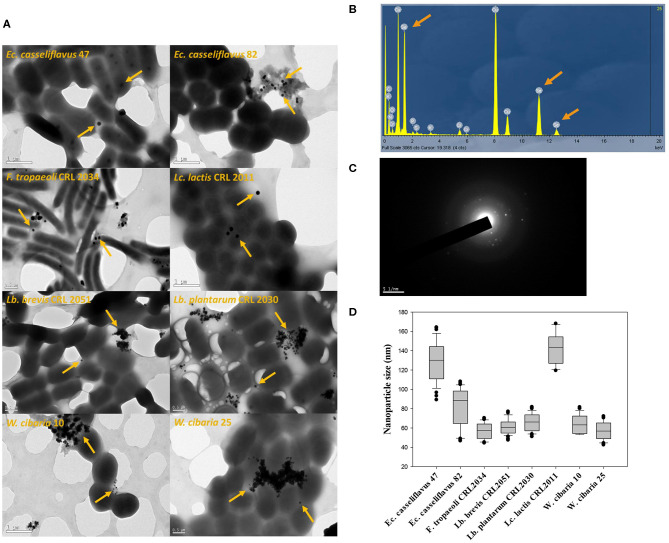
TEM images and XEDS analysis obtained for samples of eight selected strains grown in MRS/MRSf supplemented with 5 ppm Se at 30°C for 24 h. **(A)** TEM images of eight LAB strains; orange arrows indicate the presence of isolated or gathered nanoparticles. **(B)** X-Ray Energy Dispersive Spectroscopy (XEDS) analysis graph depicting energy on *X*-axis and number of counts on *Y*-axis representing elemental composition derived from selenium nanoparticles. Arrows in XEDS spectra indicate the Se emission peaks consisting of SeLα, SeKα, and SeKβ at 1.4, 11.22, and 12.49 keV, respectively. **(C)** Diffraction pattern of nanoparticles. **(D)** Nanoparticle size distribution (*n* = 100) of eight selected strains.

The eight studied strains formed spherical SeNPs (indicated with arrows) when grown in MRS/MRSf with 5 ppm Se (Figure 7A). Additionally, XEDS elementary microanalysis confirmed the presence of Se [Lα (1.4 keV), Kα (11.22 keV), and Kβ (12.49 keV)] in the visualized nanoparticles (Figure 7B). The crystalline nature of SeNPs was evidenced in the images (Figure 7C), which showed typical electron diffraction patterns for microcrystalline structures. The size distribution of the SeNPs was strain-dependent (Figure 7D). Interestingly, the two enterococci strains showed different nanoparticle sizes, whereas the size of the SeNPs of the two *W. cibaria* strains was similar. *Lc. lactis* CRL 2011 and *Ec. casseliflavus* 47 produced SeNPs with the largest diameters (125–155 nm and 110–140 nm, respectively), while the size of SeNPs of the remaining strains was between 50 and 90 nm.

### Visualization of LAB Cells and SeNPs by SEM

**Figure 8:**
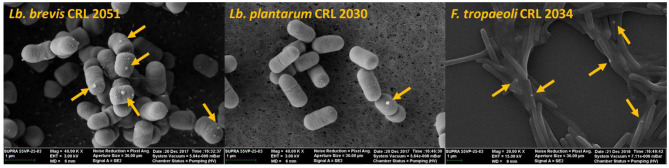
SEM microphotographs of selenized cells after 24-h incubation at 30°C in MRS or MRSf.

The effect of the presence of Se on cell morphology of the three strains that showed the highest SeCys concentrations (namely, *Lb. brevis* CRL 2051, *Lb. plantarum* CRL 2030, and *F. tropaeoli* CRL 2034) was analyzed by SEM. Neither detrimental effects on the bacterial shapes nor alterations in the cell surface were observed (Figure 8). Interestingly, the formation of SeNPs as white spheres in selenized cells was clearly noticed in the three assayed LAB.

## Discussion

Selenium is an essential trace element for human health with a recommended daily intake (RDI) of 25 to 34 μg for adults depending on age and gender according to Argentinean Food Codex (ANMAT, 2018), which is based on values established by the World Health Organization (FAO/WHO, 2004). Furthermore, it has been stated that higher Se intakes (up to 200 μg per day) can have a positive effect against free radicals, which cause age-related diseases and cancer (Alzate et al., 2010). Health benefits of Se depend on the species consumed; organic Se compounds are the least toxic and most bio-available forms for human body (Alzate et al., 2010; Palomo et al., 2014).

LAB are commonly used in the food industry worldwide due to their capacity to improve the food safety, nutritional value, flavor, and texture of fermented food products (Hugenholtz et al., 2002). In addition, functional LAB are able to increase the bioavailability of nutrients and to reduce toxic components and anti-nutritive factors present in raw materials (Tamang et al., 2016). Interestingly, some LAB can transform inorganic Se into seleno-amino acids (i.e., SeMet, SeCys, and SeMeCys), SeNPs, and/or volatile Se compounds (Pophaly et al., 2014; Javed et al., 2015; Moreno-Martin et al., 2017; Pescuma et al., 2017). Se-enrichment of bacterial cells with non-toxic Se forms could be an interesting alternative for formulating Se dietary supplements or Se enriched-containing foods.

In this work, we exhaustively studied the Se metabolism by ninety-six fruit- and flower-origin LAB strains. The bacterial capacity to grow in the presence of 5 ppm Se and to accumulate and reduce selenite producing either seleno-amino acids, volatile Se compounds, and/or SeNPs were evaluated. Furthermore, the GR enzyme activity as well as the presence of genes involved in Se metabolism were also analyzed.

When the assayed strains were grown in solid medium, all colonies displayed a time-dependent color change from white to orange-reddish. A similar behavior was observed in a *Pseudomonas alcaliphila* strain by Zhang et al. (2011). This characteristic red color is due to the excitation of the surface plasmon vibrations of the monoclinic Se particles (Lin and Wang, 2005).

As a first approach to study Se metabolism, we quantified the remaining sodium selenite in bacterial culture supernatants to indirectly measure the reductive activity on selenite oxyanion by the studied microorganisms. The 96 assayed bacteria resisted and biotransformed Se as a mechanism of cellular detoxification, as previously proposed by Lampis et al. (2014). Using this method, eight strains belonging to different genera achieved 80% selenite removal; this capacity was species- and strain-dependent. In this work, the ability of a wide diversity of LAB isolated from fruits and fruit flowers to reduce sodium selenite is shown in contrast to previous reports that were performed using strains of dairy or human origin.

Considering the Se RDI and in view of future applications of selenized cells as starter cultures for the preparation of Se-enriched functional foods, amounts of 5 ppm Se were used in our studies. All strains could grow in MRS or MRSf medium supplemented with Se although growth alteration due to the presence of this micronutrient was strain-dependent. Within the *Fructobacillus* genus, the strains belonging to the *tropaeoli* species were not affected by the presence of Se, highlighting the capability of this particular species to resist to its presence. The concentration of 5 ppm Se was used in other studies with different LAB strains. Pescuma et al. (2017) reported that *Lb. reuteri* CRL 1101 and *Lb. acidophilus* CRL 636 could grow and resist the presence of 5 ppm Se, and that the former strain was the most resistant one showing only 1 log CFU/ml decrease in cell viability after 24 h of incubation. Similar results were obtained by Lamberti et al. (2011) who reported that 4.38 ppm Se were needed to observe the same decrease in the cell viability of *Lb. reuteri* Lb2 BM. The viability of LAB in the presence of Se was studied in several works and it resulted to be dependent on the LAB used. Interestingly, the selected strains in our work were able to accumulate intracellularly between 24 and 50% of the added Se (5 ppm) after 24 h of incubation. Similarly, Palomo-Siguero et al. (2016) showed that a *Lb. bulgaricus* strain could accumulate 60% of the added Se (10 ppm) after 24 h of incubation, while Pescuma et al. (2017) found that *Lb. acidophilus* CRL 636 and *Lb. reuteri* CRL 1101 accumulated Se intracellularly, noting that the amounts increased with the incubation time; the *Lb. reuteri* strain accumulated 78% of added Se after 24 h. Chen et al. (2019) reported a 2 ppm Se accumulation by a *Lb. plantarum* strain, a similar value obtained by our strain *Lb. plantarum* CRL 2030, which showed a Se accumulation of 1.96 ± 0.05 ppm.

Noteworthy, SeCys (the most bio-available Se form) was the only Se amino acid found in the LAB cells analyzed in this work. Other authors reported that *Lactobacillus* strains could biotransform selenite into SeCys and SeMet (Palomo-Siguero et al., 2016; Pescuma et al., 2017), while only SeMet was observed for a *Bifidobacteium animalis* strain (Zhang et al., 2009) and yeast, such as *Saccharomyces cerevisiae* (Ruiz Encinar et al., 2003). The incorporation of SeCys into selenoproteins in animals and bacteria is done through a process directed by a UGA codon while SeMet is non-specifically incorporated into proteins in place of methionine during protein synthesis. Hence, SeMet is not as readily accessible as SeCys for further metabolism (Alzate et al., 2008). This fact should be considered when nutraceuticals or fermented foods are formulated using microorganisms since SeCys is the main seleno-amino acid for man and animal metabolism (Zhu et al., 2000).

As SeCys_2_ and SeMetO (Selenomethionine–Se-oxide) co-elute when a PRP-X100 anion exchange HPLC column is used, their clear discrimination is not possible (Palomo-Siguero et al., 2016); thus, the use of a second chromatographic procedure is mandatory to accurately identify Se species. Palomo-Siguero et al. (2016) and Pescuma et al. (2017) successfully used a reversed-phase Zorbax C8 column for the correct separation of Se species and proper identification of SeCys_2_. Nevertheless, one of the main problems related to the identification of the SeCys amino acid, is its high instability after extraction as it is expected to be oxidized to form S–Se or Se–Se bond, bridging Cys- or SeCys-containing peptides. Moreover, SeCys_2_ (the available commercial standard for SeCys detection by chromatographic methods) can co-elute with other non-retained Se species in some chromatographic procedures. The synthesis of carbamidomethyl-SeCys is thus a requisite for SeCys identification and the correct separation of SeCys from other species by chromatographic methods (Dernovics and Lobinski, 2008). In our work, a carbamidomethylation process to protect the SeCys structure was carried out prior to the enzymatic step. The carbamidomethylated derivates were separated by a reversed-phase EVO-C18 column coupled to ICP-MS and LC-ESI-MS/MS. The chromatogram obtained by LC-ICP-MS for the carbamidomethylated and enzymatically digested pellet of *F. tropaeoli* CRL 2034 showed a single peak eluting in the same retention time as CAM-SeCys. In order to identify this compound, a LC-MS/MS approach was carried out; the ionic fragmentation pattern obtained corresponded to the ionized fragments of the CAM-SeCys molecule.

Some bacteria can reduce Se salts into elemental Se (Se^0^) for cellular detoxification (Narasingarao and Häggblom, 2007; Lampis et al., 2014). SeNPs are formed as Se^0^ aggregates alone or in combination with exopolysaccharides and proteins. Formation of SeNPs by the eight studied LAB was confirmed by TEM images. Since surface area-to-volume ratio increases when decreasing particle size, smaller SeNPs have greater biological activity, including anti-hydroxyl radical property and protective effect against DNA oxidation (Zhang et al., 2011). On the other hand, the absorption of NPs smaller than 100 nm in the gastrointestinal tract is 15 to 250 times higher than that of larger-sized NPs (Hosnedlova et al., 2018). Interestingly, the size of SeNPs of the studied strains were between 50 and 90 nm except for the strains *Lc. lactis* CRL 2011 and *Ec. casseliflavus* 47, which produced SeNPs with larger diameters (125–155 nm and 110–140 nm, respectively). The production of SeNPs by LAB has been studied elsewhere (Eszenyi et al., 2011; Sasidharan and Balakrishnaraja, 2014; Visha et al., 2015; Moreno-Martin et al., 2017; Pescuma et al., 2017) showing also NP sizes between 40 and 500 nm depending on the strain used. Contrary to the majority of biosynthetic SeNPs like those reported by Moreno-Martin et al. (2017), the nanoparticles produced by our strains were microcrystalline. Also, Visha et al. (2015) reported a crystalline structure for the SeNPs produced by a *Lb. acidophilus* strain; these differences demonstrate the particle diversity produced by LAB. SeNPs are less toxic than inorganic Se; moreover, their capacity to diminish cancer has been studied *in vivo* and *in vitro* (Yazdi et al., 2013; Lopez-Heras et al., 2014). Yazdi et al. (2012) reported that a *Lb. plantarum* strain enriched with SeNPs could elevate INF-γ, TNF-α, and IL-2 levels as well as natural killer cell activity in a murine model. Moreover, a SeNP-enriched *Lb. brevis* strain inhibited liver metastasis in metastatic mouse breast cancer (Yazdi et al., 2013). Interestingly, it has been reported that biogenic SeNPs possessed antimicrobial properties and that, when administered alone or in combination with antibiotics, they inhibit growth of multi-resistant bacteria and can disaggregate biofilms produced by these pathogens (Cremonini et al., 2016). Furthermore, Se-enriched probiotics improved lipid metabolism, antioxidant status, and histopathological lesions induced by a fat-rich diet in an obesity model in mice (Nido et al., 2016).

Some microorganisms can reduce Se oxyanions (Yee et al., 2014), playing a fundamental role in the recycling and transformation of Se through redox and methylation reactions. Microorganisms living in Se-rich environments are usually able to convert inorganic Se compounds into volatile compounds. These microorganisms can secrete methyltransferases stimulating the emission of DMSe (Javed et al., 2015). Volatile Se compounds have a strong odor and can contribute to unpleasant flavors in foods; thus, bacteria producing these compounds may be unsuitable for their use in the food industry (Michalke et al., 2000). Our results showed that among the eight selected strains, only *Ec. casseliflavus* 47 and 82 were able to form DMDSe and DMSe, respectively, although in scarce amounts. Pophaly et al. (2014) reported the formation of the same compounds by the strains *Streptococcus salivarius* K12, *Lb. rhamnosus* 67B, *Lb. acidophilus* L10, and *B. lactis* LAFTI B94 when the cultures were previously enriched with SeMet, while formation of DMSe, DMDSe, and Se^0^ was observed when selenite enrichment was done.

In many bacteria and eukaryotes, reduced glutathione (GSH) is a prime candidate as thiol compound source because GSH is the most abundant low-molecular-weight thiol in these organisms. Selenite can react with several thiols producing seleno-trisulfide derivatives. When GSH reacts with selenite, selenodiglutathione (GSSeSG) is produced; this compound is a key intermediate in the Se metabolic pathway, leading to the conversion of inorganic Se into bioactive selenocompounds such as Se^0^ (SeNPs) (Ogasawara et al., 2005) or selenophosphate through the selenophosphate synthetase enzyme activity (Seale, 2019). The enzyme GR catalyzes NADPH-dependent reduction of glutathione and has an important role in cell defense against oxygen stress by maintaining a high intracellular GSH/GSSG level (Jänsch et al., 2007). Chung and Maines (1981) reported that Se could increase the activities of γ-glutamyl cysteine synthetase, the first and rate-limiting enzyme in GSH biosynthesis, and GR, which catalyzes the reduction of GSSG to GSH. The increase in the activities of these enzymes was observed 24 h after Se (10 and 20 pmol/kg) administration. This finding coincides with our results in which the GR activity presented a two- to six-fold increase when the strains were grown in the presence of Se after 24 h of incubation, indicating an active Se metabolism; an opposite behavior was seen for the *F. tropaeoli* strain.

On the other hand, the enzyme SCL is able to decompose SeCys into Se^0^ and alanine; this enzyme can also mobilize Se into SeCys for selenophosphate synthesis necessary for producing SeCys-tRNA, the precursor of SeCys and selenoproteins (Ogasawara et al., 2005; Lamberti et al., 2011). The SCL activity was detected in several bacterial species, such as *Pseudomonas alkanolytica, Alcaligenes viscolactis*, and *Escherichia freundii* (Chocat et al., 1983). In bacteria, SCL activity has been related to three enzymes, the *Azobacter vinelandii* Nifs-like protein, and the *E. coli* CsdA and CsdB cysteine desulfurases (Lacourciere et al., 2000). Moreover, a SCL/cysteine desulfurase regulated by selenite levels was identified in a probiotic *Lb. reuteri* strain by Lamberti et al. (2011). These enzymes can decompose L-cysteine and L-SeCys into alanine and elemental sulfur and selenium, respectively (Seale, 2019). Both enzymes GR and SCL have been detected in some LAB strains (Lamberti et al., 2011; Pusztahelyi et al., 2015); however, Se metabolism has been studied mainly in dairy- or human gut-origin LAB (Zhang et al., 2009; Mangiapane et al., 2014; Palomo et al., 2014; Saini et al., 2014; Deng et al., 2015; Pescuma et al., 2017; Gomez-Gomez et al., 2019). The gene *GshR* coding for GR was detected in the eight analyzed strains, while *Scl* could be amplified in all strains except for the *Lactobacillus* strains with the primers used. The presence of these genes supports the capability of these strains to metabolize Se and to produce SeNPs and SeCys.

In this work, we studied nine *Fructobacillus* strains that belong to a particular group of bacteria called FLAB. This group, which was described a decade ago and consists of five species of *Fructobacillus* and a few *Lactobacillus* species, shares some unique characteristics including poor growth on glucose, the preference of oxygen, and the need of an external electron acceptor to grow, such as fructose, which is the optimum substrate for growth (Endo et al., 2009). It is thus not surprising to find FLAB in fructose-rich environments, including flowers, fruits, fermented foods derived from fruits and even guts of insects that feed on plants rich in fructose (Endo and Salminen, 2013). The genomic level of FLAB changed to adapt to these different types of environments; further studies are required to explore their beneficial properties in animals and humans and their applications in the food industry (Endo et al., 2018). Among the nine *Fructobacillus* strains isolated from fig (7 strains), custard apple (1), and khaki (1) studied in this work, we selected the fig-isolate *F. tropaeoli* CRL 2034, which presented the largest selenite percentage removal from the culture medium. This strain could accumulate Se intracellularly and produce SeNPs and SeCys in large amounts, while the genes encoding for GR and SCL enzymes were found in the genome; nevertheless, the GR activity decreased when the strain was grown in the presence of Se. This could be due to differences in the requirements and metabolism of this group of LAB. The unusual growth characteristics of FLAB are a consequence of the incomplete gene *adhE* encoding a bi-functional alcohol/acetaldehyde dehydrogenase, which results in the imbalance of NAD/NADH and the requirement of additional electron acceptors to metabolize glucose such as oxygen, fructose, and pyruvate (Endo et al., 2018). It is likely that the NAD/NADH imbalance could alter the normal Se metabolism. Also, FLAB could use Se as an electron acceptor in a GSH/GR-independent manner reflecting on the reduction of GR activity; however, further studies need to be done to better understand Se metabolism in this bacterial group.

Interestingly, LAB able to biotransform Se could be established in the intestine and biotransform the inorganic Se ingested by the diet or supplements into organic Se (i.e., seleno-amino acids); this fact is feasible if the strains survive the human gastrointestinal conditions. Martinez et al. (2019) showed that the fruit-origin strains *Lb. brevis* CRL 2051 and *F. tropaeoli* CRL 2034 could survive the *in vitro* gastrointestinal tract conditions when present in a fermented fruit juice-milk beverage. The *Fructobacillus* strain preferred fructose to glucose to grow and produced the low calorie sugar mannitol in an equal amount of that of fructose consumed; mannitol may provide health beneficial effects by acting as antioxidant and low-calorie sweetener applicable for diabetic patients (Ortiz et al., 2015). In this study, the presence of selenite did not affect cell survival of *Lb. brevis* CRL 2051 but improved the *Fructobacillus* strain viability after a 28-day storage period (Martinez et al., 2019). Remarkably, selenized cells of the *Fructobacillus* strain were more resistant to the incubation with digestive enzymes than non-selenized bacteria, indicating that Se accumulation may confer a selective advantage to tolerate digestion. In contrast, no effect was detected with the *Lb. brevis* strain. These results demonstrate that *Lb. brevis* CRL 2051 and *F. tropaeoli* CRL 2034 may be used as Se-enriched strains in the industry of functional foods. Also, Pescuma et al. (2017) reported that the growth of a selenized *Lb. reuteri* strain was not affected by the presence of bile salts in the culture medium while a negative effect on the growth of non-selenized strain was noticed. Similar results were reported for *Lb. reuteri* Lb2BM by Mangiapane et al. (2014).

Nowadays, a worldwide tendency to consume fruit and vegetable fermented products exists due to the high incidence of milk allergy/intolerance, the search for cholesterol-free foods, and vegan habits (Di Cagno et al., 2010), among others. In this context, the ability of the studied strains to biotransform and accumulate Se is highly relevant since they can better grow in fruit matrixes than bacteria coming from different niches. Moreover, the selected strains produced only the Se amino acid SeCys, which is more bioavailable and has no toxic effect as it has been observed for SeMet, when consumed in high concentrations. In addition, these strains produced small-size SeNPs, which may be better absorbed in the gastrointestinal tract than bigger size ones.

## Conclusions

Eight LAB strains were selected for their ability to grow, resist, and biotransform inorganic Se into organic forms. These microorganisms could accumulate Se intracellularly, produce SeNPs and incorporate SeCys, which is the most bioavailable Se form. In this work, the Se metabolism was extensively studied in selected strains. Furthermore, the ability of two *Enterococcus* strains to produce volatile Se compounds is reported in this study. Moreover, differences in Se metabolism were observed for a *Fructobacillus* strain for which GR activity was inhibited in the presence of Se, indicating that other mechanisms in selenite reduction are involved. Our findings suggest that *Lb brevis* CRL 2051, *Lb. plantarum* CRL 2030, and *F. tropaeoli* CRL 2034 could be used for the development of nutraceuticals or be used as starter cultures for the bio-enrichment of fermented (fruit) beverages with SeCys and SeNPs.

## Data Availability Statement

The raw data supporting the conclusions of this article will be made available by the authors, without undue reservation.

## Author Contributions

FGM conducted the experimental work, analyzed the results, and wrote the manuscript. GM-M conducted with FGM some experimental determinations and analyzed some of the results. MP directed and designed the work, and wrote and revised the manuscript. YM-A directed part of the work and revised the manuscript. FM directed the work and revised the manuscript. All authors read and approved the submitted version.

## Conflict of Interest

The authors declare that the research was conducted in the absence of any commercial or financial relationships that could be construed as a potential conflict of interest.

## References

[B1] Alzate A. Fernández-Fernández A. Pérez-Conde M. Gutiérrez A. Cámara C. (2008). Comparison of biotransformation of inorganic selenium by Lactobacillus and Saccharomyces in lactic fermentation process of yogurt and kefir. J. Agric. Food Chem. 56, 8728–8736. 10.1021/jf8013519 18729458

[B2] Alzate A. Pérez-Conde M. Gutiérrez A. Cámara C. (2010). Selenium-enriched fermented milk: a suitable dairy product to improve selenium intake in humans. Int. Dairy J. 20, 761–769. 10.1016/j.idairyj.2010.05.007

[B3] ANMAT (2018). Codigo Alimentario Argentino. Capítulo XVII: Alimentos de régimen o dietéticos. Available online at: https://www.argentina.gob.ar/anmat/codigoalimentario (accessed February 20, 2020).

[B4] Brown T. A. (1995). Purification of DNA from Living Cells, in Gene Cloning: An Introduction. Manchester: Stanley Thornes, Chapman & Hall, 27–51.

[B5] Chen Y. Li Q. Xia C. Yang F. Xu N. Wu Q.. (2019). Effect of selenium supplements on the antioxidant activity and nitrite degradation of lactic acid bacteria. World J. Microbiol. Biotechnol. 35:61. 10.1007/s11274-019-2609-x 30919142

[B6] Chocat P. Esaki N. Nakamura T. Tanaka H. Soda K. (1983). Microbial distribution of selenocysteine lyase. J. Bacteriol. 156, 455–457. 10.1128/JB.156.1.455-457.1983 6225771 PMC215108

[B7] Chung A-S. Maines M. D. (1981). Effect of selenium on glutathione metabolism. Induction of γ-glutamylcysteine synthetase and glutathione reductase in the rat liver. Biochem. Pharmacol. 30, 3217–3223. 10.1016/0006-2952(81)90521-9 6119089

[B8] Cremonini E. Zonaro E. Donini M. Lampis S. Boaretti M. Dusi S.. (2016). Biogenic selenium nanoparticles: characterization, antimicrobial activity and effects on human dendritic cells and fibroblasts. Microb. Biotechnol. 9, 758–771. 10.1111/1751-7915.12374 27319803 PMC5072192

[B9] Deng Y. Man C. Fan Y. Wang Z. Li L. Ren H. (2015). Preparation of elemental selenium-enriched fermented milk by newly isolated *Lactobacillus brevis* from kefir grains. Int. Dairy J. 44, 31–36. 10.1016/j.idairyj.2014.12.008

[B10] Dernovics M. Lobinski R. (2008). Characterization of the selenocysteine-containing metabolome in selenium-rich yeast Part II. on the reliability of the quantitative determination of selenocysteine. J. Anal. At. Spectrom. 23, 744–751. 10.1039/b716140a

[B11] Dhanjal S. Cameotra S. S. (2010). Aerobic biogenesis of selenium nanospheres by *Bacillus cereus* isolated from coalmine soil. Microb. Cell Fact. 9:52. 10.1186/1475-2859-9-52 20602763 PMC2909957

[B12] Di Cagno R. Cardinali G. Minervini G. Antonielli L. Rizzello C. G. Ricciuti P.. (2010). Taxonomic structure of the yeasts and lactic acid bacteria microbiota of pineapple (*Ananas comosus L. Merr.)* and use of autochthonous starters for minimally processing. Food Microbiol. 27, 381–389. 10.1016/j.fm.2009.11.012 20227603

[B13] Endo A. Futagawa-Endo Y. Dicks L. M. (2009). Isolation and characterization of fructophilic lactic acid bacteria from fructose-rich niches. Syst. Appl. Microbiol. 32, 593–600. 10.1016/j.syapm.2009.08.002 19733991

[B14] Endo A. Maeno S. Tanizawa Y. Kneifel W. Arita M. Dicks L.. (2018). Fructophilic lactic acid bacteria, a unique group of fructose-fermenting microbes. Appl. Environ. Microbiol. 84, e01290–e01218. 10.1128/AEM.01290-18 30054367 PMC6146980

[B15] Endo A. Salminen S. (2013). Honeybees and beehives are rich sources for fructophilic lactic acid bacteria. Syst. Appl. Microbiol. 36, 444–448. 10.1016/j.syapm.2013.06.002 23845309

[B16] Eszenyi P. Sztrik A. Babka B. Prokisch J. (2011). Elemental, nano-sized (100-500 nm) selenium production by probiotic lactic acid bacteria. Int. J. Biosci. Biochem. Bioinform. 1:148 10.7763/IJBBB.2011.V1.27

[B17] FAO/WHO, (2004). Vitamin and Mineral Requirements in Human Nutrition. Chapter 10, Selenium. Bangkok: World Health Organization Second edition, 194–216

[B18] Gomez-Gomez B. Perez-Corona T. Mozzi F. Pescuma M. Madrid Y. (2019). Silac-based quantitative proteomic analysis of *Lactobacillus reuteri* CRL 1101 response to the presence of selenite and selenium nanoparticles. J. Proteomics 195, 53–65. 10.1016/j.jprot.2018.12.025 30593931

[B19] Hosnedlova B. Kepinska M. Skalickova S. Fernandez C. Ruttkay-Nedecky B. Peng Q.. (2018). Nano-selenium and its nanomedicine applications: a critical review. Int. J. Nanomed. 13, 2107–2128. 10.2147/IJN.S157541 29692609 PMC5901133

[B20] Hugenholtz J. Sybesma W. Groot M. N. Wisselink W. Ladero V. Burgess K.. (2002). Metabolic engineering of lactic acid bacteria for the production of nutraceuticals. Antonie Van Leeuwenhoek. 82, 217–235. 10.1007/978-94-017-2029-8_13 12369189

[B21] Jänsch A. Korakli M. Vogel R. F. Gänzle M. G. (2007). Glutathione reductase from *Lactobacillus sanfranciscensis* DSM20451T: contribution to oxygen tolerance and thiol exchange reactions in wheat sourdoughs. Appl. Environ. Microbiol. 73, 4469–4476. 10.1128/AEM.02322-06 17496130 PMC1932818

[B22] Javed S. Sarwar A. Tassawar M. Faisal M. (2015). Conversion of selenite to elemental selenium by indigenous bacteria isolated from polluted areas. Chem. Spec. Bioavailab. 27, 162–168. 10.1080/09542299.2015.1112751

[B23] Kessi J. Ramuz M. Wehrli E. Spycher M. Bachofen R. (1999). Reduction of selenite and detoxification of elemental selenium by the phototrophic bacterium *Rhodospirillum rubrum*. Appl. Environ. Microbiol. 65, 4734–4740. 10.1128/AEM.65.11.4734-4740.1999 10543779 PMC91637

[B24] Lacourciere G. M. Mihara H. Kurihara T. Esaki N. Stadtman T. C. (2000). *Escherichia coli* NifS-like proteins provide selenium in the pathway for the biosynthesis of selenophosphate. J. Biol. Chem. 275, 23769–23773. 10.1074/jbc.M000926200 10829016

[B25] Lamberti C. Mangiapane E. Pessione A. Mazzoli R. Giunta C. Pessione E. (2011). Proteomic characterization of a selenium-metabolizing probiotic *Lactobacillus reuteri* Lb2 BM for nutraceutical applications. Proteomics 11, 2212–2221. 10.1002/pmic.201000747 21548091

[B26] Lampis S. Zonaro E. Bertolini C. Bernardi P. Butler C. S. Vallini G. (2014). Delayed formation of zero-valent selenium nanoparticles by *Bacillus mycoides* SeITE01 as a consequence of selenite reduction under aerobic conditions. Microb. Cell Fact. 13:35. 10.1186/1475-2859-13-35 24606965 PMC3975340

[B27] Lin Z-H. Wang C. C. (2005). Evidence on the size-dependent absorption spectral evolution of selenium nanoparticles. Mater. Chem. Phys. 92, 591–594. 10.1016/j.matchemphys.2005.02.023

[B28] Lopez-Heras I. Sanchez-Diaz R. Anunciação D. S. Madrid Y. Luque-Garcia J. L. Camara C. (2014). Effect of chitosan-stabilized selenium nanoparticles on cell cycle arrest and invasiveness in hepatocarcinoma cells revealed by quantitative proteomics. J. Nanomed. Nanotechnol. 5:1 10.4172/2157-7439.1000226

[B29] Mangiapane E. Lamberti C. Pessione A. Galano E. Amoresano A. Pessione E. (2014). Selenium effects on the metabolism of a Se-metabolizing *Lactobacillus reuteri*: analysis of envelope-enriched and extracellular proteomes. Mol. Biosyst. 10, 1272–1280. 10.1039/C3MB70557A 24481235

[B30] Martinez F. G. Cuencas Barrientos M. E. Mozzi F. Pescuma M. (2019). Survival of selenium-enriched lactic acid bacteria in a fermented drink under storage and simulated gastro-intestinal digestion. Food Res. Int. 123, 115–124. 10.1016/j.foodres.2019.04.057 31284959

[B31] Michalke K. Wickenheiser E. Mehring M. Hirner A. Hensel R. (2000). Production of volatile derivatives of metal (loid) s by microflora involved in anaerobic digestion of sewage sludge. Appl. Environ. Microbiol. 66, 2791–2796. 10.1128/AEM.66.7.2791-2796.2000 10877769 PMC92074

[B32] Moreno-Martin G. Pescuma M. Pérez-Corona T. Mozzi F. Madrid Y. (2017). Determination of size and mass-and number-based concentration of biogenic SeNPs synthesized by lactic acid bacteria by using a multimethod approach. Anal. Chim. Acta 992, 34–41. 10.1016/j.aca.2017.09.033 29054148

[B33] Moreno-Martin G. Sanz-Landaluze J. León-Gonzalez M. E. Madrid Y. (2019). *In-vivo* solid phase microextraction for quantitative analysis of volatile organoselenium compounds in plants. Anal. Chim. Acta 1081, 72–80. 10.1016/j.aca.2019.06.061 31446967

[B34] Mounicou S. Vonderheide A. P. Shann J. R. Caruso J. A. (2006). Comparing a selenium accumulator plant (*Brassica juncea*) to a nonaccumulator plant (*Helianthus annuus*) to investigate selenium-containing proteins. Anal. Bioanal. Chem. 386, 1367–1378. 10.1007/s00216-006-0707-8 16933129

[B35] Narasingarao P. Häggblom M. M. (2007). Identification of anaerobic selenate-respiring bacteria from aquatic sediments. Appl. Environ. Microbiol. 73, 3519–3527. 10.1128/AEM.02737-06 17435005 PMC1932684

[B36] Nido S. A. Shituleni S. A. Mengistu B. M. Liu Y. Khan A. Z. Gan F.. (2016). Effects of selenium-enriched probiotics on lipid metabolism, antioxidative status, histopathological lesions, and related gene expression in mice fed a high-fat diet. Biol. Trace Elem. Res. 171, 399–409. 10.1007/s12011-015-0552-8 26546553

[B37] Ogasawara Y. Lacourciere G. M. Ishii K. Stadtman T. C. (2005). Characterization of potential selenium-binding proteins in the selenophosphate synthetase system. Proc. Natl. Acad. Sci.U.S.A. 102, 1012–1016. 10.1073/pnas.0409042102 15653770 PMC545862

[B38] Ortiz M. E. Raya R. R. Mozzi F. (2015). Efficient mannitol production by wild-type *Lactobacillus reuteri* CRL 1101 is attained at constant pH using a simplified culture medium. Appl. Microbiol. Biotechnol. 99, 8717–8729. 10.1007/s00253-015-6730-y 26084891

[B39] Palomo M. Gutiérrez A. M. Pérez-Conde M. C. Cámara C. Madrid Y. (2014). Se metallomics during lactic fermentation of Se-enriched yogurt. Food Chem. 164, 371–379. 10.1016/j.foodchem.2014.05.007 24996347

[B40] Palomo-Siguero M. Gutiérrez A. M. A. Pérez-Conde C. Madrid Y. (2016). Effect of selenite and selenium nanoparticles on lactic bacteria: a multi-analytical study. Microchem. J. 126, 488–495. 10.1016/j.microc.2016.01.010

[B41] Palomo-Siguero M. Madrid Y. (2017). Exploring the behavior and metabolic transformations of SeNPs in exposed lactic acid bacteria. Effect of nanoparticles coating agent. Int. J. Mol. Sci. 18:1712. 10.3390/ijms18081712 28783048 PMC5578102

[B42] Pedrero Z. Madrid Y. Cámara C. (2006). *Selenium species* bioaccessibility in enriched radish (*Raphanus sativus*): a potential dietary source of selenium. J. Agric. Food Chem. 54, 2412–2417. 10.1021/jf052500n 16536627

[B43] Pescuma M. Gomez-Gomez B. Perez-Corona T. Font G. Madrid Y. Mozzi F. (2017). Food prospects of selenium enriched-*Lactobacillus acidophilus* CRL 636 and *Lactobacillus reuteri* CRL 1101. J. Funct. Foods 35, 466–473. 10.1016/j.jff.2017.06.009

[B44] Pophaly S. Poonam S. Pophaly S. Kapila S. Nanda D. Tomar S.. (2017). Glutathione biosynthesis and activity of dependent enzymes in food-grade lactic acid bacteria harbouring multidomain bifunctional fusion gene (gshF). J. Appl. Microbiol. 123, 194–203. 10.1111/jam.13471 28403558

[B45] Pophaly S. D. Singh P. Kumar H. Tomar S. K. Singh R. (2014). Selenium enrichment of lactic acid bacteria and bifidobacteria: a functional food perspective. Trends Food Sci. Technol. 39, 135–145. 10.1016/j.tifs.2014.07.006

[B46] Pusztahelyi T. Kovács S. Pócsi I. Prokisch J. (2015). Selenite-stress selected mutant strains of probiotic bacteria for Se source production. J. Trace Elem. Med. Biol. 30, 96–101. 10.1016/j.jtemb.2014.11.003 25524403

[B47] Rayman M. P. (2012). Selenium and human health. Lancet 379, 1256–1268. 10.1016/S0140-6736(11)61452-9 22381456

[B48] Ruiz Encinar J. Ouerdane L. Buchmann W. Tortajada J. Lobinski R. Szpunar J. (2003). Identification of water-soluble selenium-containing proteins in selenized yeast by size-exclusion-reversed-phase HPLC/ICPMS followed by MALDI-TOF and electrospray Q-TOF mass spectrometry. Anal. Chem. 75, 3765–3774. 10.1021/ac034103m 14572042

[B49] Ruiz Rodríguez L. G. Aller K. Bru E. De Vuyst L. Hébert E. M. Mozzi F. (2017). Enhanced mannitol biosynthesis by the fruit origin strain *Fructobacillus tropaeoli* CRL 2034. Appl. Microbiol. Biotechnol. 101, 6165–6177. 10.1007/s00253-017-8395-1 28674850

[B50] Ruiz Rodriguez L. G. Mohamed F. Bleckwedel J. Medina R. B. De Vuyst L. Hebert E. M.. (2019). Diversity and functional properties of lactic acid bacteria isolated from wild fruits and flowers present in Northern Argentina. Front. Microbiol. 10:1091. 10.3389/fmicb.2019.01091 31164879 PMC6536596

[B51] Saini K. Tomar S. K. Sangwan V. Bhushan B. (2014). Evaluation of lactobacilli from human sources for uptake and accumulation of selenium. Biol. Trace Elem. Res. 160, 433–436. 10.1007/s12011-014-0065-x 25022245

[B52] Sasidharan S. Balakrishnaraja R. (2014). Comparison studies on the synthesis of selenium nanoparticles by various microorganisms. Int. J. Pure Appl. Biosci. 2, 112–117.

[B53] Seale L. A. (2019). Selenocysteine β-Lyase: biochemistry, regulation and physiological role of the selenocysteine decomposition enzyme. Antioxidants 8:357. 10.3390/antiox8090357 31480609 PMC6770646

[B54] Tamang J. P. Shin D.-H. Jung S.-J. Chae S.-W. (2016). Functional properties of microorganisms in fermented foods. Front. Microbiol. 7:578. 10.3389/fmicb.2016.00578 27199913 PMC4844621

[B55] Visha P. Nanjappan K. Selvaraj P. Jayachandran S. Elango A. Kumaresan G. (2015). Biosynthesis and structural characteristics of selenium nanoparticles using *Lactobacillus acidophilus* bacteria by wet sterilization process. Int. J. Adv. Vet. Sci. Technol. 4, 178–183. 10.23953/cloud.ijavst.183

[B56] Yazdi M. Mahdavi M. Kheradmand E. Shahverdi A. (2012). The preventive oral supplementation of a selenium nanoparticle-enriched probiotic increases the immune response and lifespan of 4T1 breast cancer bearing mice. Arzneimittelforschung 62, 525–531. 10.1055/s-0032-1323700 22945771

[B57] Yazdi M. H. Mahdavi M. Setayesh N. Esfandyar M. Shahverdi A. R. (2013). Selenium nanoparticle-enriched *Lactobacillus brevis* causes more efficient immune responses *in vivo* and reduces the liver metastasis in metastatic form of mouse breast cancer. DARU J.Pharm. Sci. 21:33. 10.1186/2008-2231-21-33 23631392 PMC3658950

[B58] Yee N. Choi J. Porter A. W. Carey S. Rauschenbach I. Harel A. (2014). Selenate reductase activity in *Escherichia coli* requires Isc iron–sulfur cluster biosynthesis genes. FEMS Microbiol. Lett. 361, 138–143. 10.1111/1574-6968.12623 25307727

[B59] Zhang B. Zhou K. Zhang J. Chen Q. Liu G. Shang N. (2009). Accumulation and species distribution of selenium in Se-enriched bacterial cells of the *Bifidobacterium animalis* 01. Food Chem. 115, 727–734. 10.1016/j.foodchem.2008.12.006

[B60] Zhang W. Chen Z. Liu H. Zhang L. Gao P. Li D. (2011). Biosynthesis and structural characteristics of selenium nanoparticles by *Pseudomonas alcaliphila*. Colloids Surf. B Biointerfaces 88, 196–201. 10.1016/j.colsurfb.2011.06.031 21752611

[B61] Zhu Z. Jiang W. Ganther H. E. Ip C. Thompson H. J. (2000). Activity of Se-allylselenocysteine in the presence of methionine γ-lyase on cell growth, DNA integrity, apoptosis, and cell-cycle regulatory molecules. Mol. Carcinog. 29, 191–197. 10.1002/1098-2744(200012)29:4<191::aid-mc1000>3.0.co;2-7 11170256

