# Supplementary material for: Biotransformation of Selenium by Lactic Acid Bacteria: Formation of Seleno-Nanoparticles and Seleno-Amino Acids
Source: Front Bioeng Biotechnol. 2020 Jun 12;8:506. doi: 10.3389/fbioe.2020.00506 (PMC7303280; doi:10.3389/fbioe.2020.00506)
Supplement: Supplementary file 1 [file Table_1.pdf]

Table S1.Growth parameters of 96 LAB strains isolated from wild fruits and flowers from northern Argentina. Color scale represents different values, red meaning higher and green lower ones

LAB strains	μ max		ΔOD600 8h		ΔOD600 24h	
	Control	Se	Control	Se	Control	Se
Lc. lactis subsp. lactis CRL 2009	0.62	0.17	0.85	0.26	0.91	0.78
Lc. lactis subsp. lactis CRL 2010	0.50	0.46	0.82	0.66	0.92	0.82
Lc. lactis subsp. lactis CRL 2011	0.57	0.53	0.81	0.67	0.94	0.87
Lc. lactis subsp. cremoris CRL 2029	0.51	0.50	0.47	0.46	0.75	0.65
Lc. lactis CRL 2025	0.21	0.22	1.08	0.92	1.08	0.99
W. cibaria 10	0.46	0.40	0.66	0.55	1.27	1.26
W. cibaria 11	0.42	0.43	0.75	0.55	1.17	1.22
W. cibaria 12	0.34	0.37	0.81	0.66	1.21	1.21
W. cibaria 25	0.31	0.33	1.10	0.94	1.45	1.44
W. fabalis 14	0.42	0.44	0.35	0.37	0.72	0.82
W. minor 15	0.31	0.29	0.39	0.36	1.14	1.05
W. minor CRL 2099	0.32	0.29	0.29	0.27	1.01	0.95
W. minor 17	0.29	0.25	0.27	0.25	1.05	1.03
W. minor 18	0.33	0.32	0.51	0.50	1.15	1.06
W. minor 19	0.32	0.32	0.49	0.45	1.15	1.10
W. minor 20	0.37	0.36	0.37	0.35	1.22	1.17
W. minor 21	0.38	0.38	0.96	0.86	1.46	1.41
W. minor 22	0.35	0.34	0.56	0.53	1.16	1.10
W. minor 23	0.18	0.17	0.33	0.34	1.49	1.49
Leuc. mesenteroides subsp. mesenteroides CRL 2117	0.40	0.11	0.45	0.23	1.21	1.11
Leuc. mesenteroides subsp. mesenteroides CRL 2027	0.41	0.42	0.55	0.47	1.07	0.72
Leuc. mesenteroides subsp. mesenteroides CRL 2119	0.12	0.12	0.30	0.20	1.17	0.98
Leuc. mesenteroides subsp. mesenteroides CRL 2022	0.29	0.30	1.05	1.00	1.45	1.45
Leuc. mesenteroides subsp. mesenteroides CRL 2120	0.24	0.24	1.01	1.00	1.46	1.45
Leuc. mesenteroides 30	0.27	0.33	0.74	0.56	1.26	1.26
Leuc. mesenteroides CRL 1998	0.46	0.43	0.71	0.58	1.46	1.46
Leuc. mesenteroides CRL 2059	0.39	0.12	0.40	0.25	1.27	0.97
Leuc. mesenteroides CRL 2026	0.39	0.33	0.52	0.31	1.26	0.97

Leuc. mesenteroides CRL 2021	0.41	0.30	0.52	0.36	1.11	1.26
Leuc. pseudomesenteroides CRL 2023	0.28	0.28	0.83	0.70	1.30	1.20
Leuc. pseudomesenteroides CRL 1996	0.38	0.39	0.44	0.36	1.08	1.08
Leuc. pseudomesenteroides CRL 1997	0.43	0.48	0.32	0.26	0.86	0.45
Leuc. pseudomesenteroides CRL 2024	0.47	0.49	0.57	0.40	1.32	1.28
Leuc. pseudomesenteroides CRL 2028	0.38	0.40	0.94	0.85	1.34	1.25
Leuc. pseudomesenteroides 45	0.14	0.16	0.31	0.21	1.28	1.14
Leuc. pseudomesenteroides 46	0.14	0.20	0.04	0.01	0.44	0.49
Leuc. pseudomesenteroides 48	0.26	0.28	0.91	0.90	1.26	1.45
Leuc. pseudomesenteroides 49	0.41	0.39	0.55	0.57	0.94	1.09
Leuc. pseudomesenteroides CRL 1994	0.47	0.45	1.07	1.06	1.42	1.46
Leuc. pseudomesenteroides 51	0.48	0.48	0.92	0.82	1.35	1.37
Leuc. pseudomesenteroides 53	0.53	0.46	0.98	0.84	1.48	1.47
Leuc. pseudomesenteroides 54	0.50	0.49	1.15	1.06	1.45	1.46
Leuc. pseudomesenteroides 55	0.50	0.50	1.08	1.06	1.48	1.46
Leuc. pseudomesenteroides 56	0.48	0.43	1.16	1.05	1.46	1.45
Leuc. pseudomesenteroides 57	0.46	0.47	1.16	1.06	1.46	1.46
Leuc. pseudomesenteroides 58	0.45	0.43	1.15	1.05	1.45	1.45
Leuc. pseudomesenteroides 59	0.38	0.35	1.13	1.12	1.38	1.47
Leuc. pseudomesenteroides 60	0.37	0.36	0.54	0.49	1.36	1.25
Leuc. pseudomesenteroides 61	0.39	0.39	1.03	1.02	1.38	1.42
Leuc. pseudomesenteroides 62	0.39	0.38	1.07	0.97	1.37	1.37
Leuc. pseudomesenteroides 63	0.36	0.36	1.07	1.03	1.37	1.43
Leuc. pseudomesenteroides 64	0.36	0.33	1.08	1.07	1.43	1.37
Leuc. pseudomesenteroides 65	0.36	0.37	1.05	0.97	1.45	1.47
Leuc. pseudomesenteroides 66	0.36	0.33	1.08	1.07	1.48	1.47
Leuc. pseudomesenteroides 67	0.38	0.36	1.06	1.00	1.46	1.45
Leuc. pseudomesenteroides 68	0.37	0.38	1.06	0.92	1.46	1.37
Leuc. pseudomesenteroides 69	0.25	0.24	1.01	0.96	1.46	1.46
Leuc. citreum CRL 2057	0.46	0.43	0.92	0.87	1.27	1.27
Leuc. citreum CRL 2058	0.31	0.34	0.97	0.93	1.27	1.28
Lb. brevis CRL 2050	0.16	0.16	0.16	0.14	1.14	1.27
Lb. brevis CRL 2051	0.38	0.38	0.66	0.66	1.46	1.46
Lb. brevis CRL 2052	0.26	0.25	0.50	0.52	0.70	1.42
Lb. brevis CRL 2053	0.12	0.11	0.18	0.17	1.02	0.90

Lb. brevis CRL 2055	0.29	0.29	0.56	0.57	1.44	1.43
Lb. brevis CRL 2056	0.36	0.36	0.48	0.47	1.06	1.45
Lb. brevis CRL 2057	0.37	0.37	0.51	0.49	1.17	1.47
Lb. plantarum CRL 2030	0.45	0.44	1.05	1.09	1.90	1.89
Lb. rhamnosus CRL 2031	0.25	0.20	0.52	0.26	1.62	1.37
Lb. rhamnosus CRL 2049	0.10	0.09	0.51	0.35	1.61	1.40
Ec. casseliflavus 47	0.40	0.19	-0.01	0.01	0.55	0.39
Ec. casseliflavus 82	0.51	0.46	0.37	0.36	0.54	0.57
Ec. casseliflavus 83	0.43	0.37	0.43	0.29	0.66	0.62
Ec. casseliflavus 84	0.55	0.47	0.26	0.25	0.56	0.59
Ec. casseliflavus 85	0.49	0.45	0.40	0.22	0.75	0.57
Ec. casseliflavus 86	0.38	0.37	0.43	0.47	0.75	0.73
Ec. casseliflavus 87	0.34	0.38	0.44	0.48	0.78	0.78
Ec. casseliflavus 88	0.60	0.47	0.37	0.35	0.54	0.57
Ec. faecalis 89	0.44	0.40	0.66	0.57	1.18	1.19
Ec. hirae 90	0.64	0.52	0.34	0.29	0.80	0.54
Ec. hirae 91	0.45	0.43	0.60	0.45	1.04	0.84
Ec. hirae 92	0.47	0.53	0.63	0.54	1.03	0.83
Ec. hirae 93	0.43	0.52	0.50	0.44	0.94	0.73
Ec. muntii 94	0.29	0.29	0.93	0.84	1.33	1.34
Ec. faecium 95	0.37	0.38	0.43	0.35	0.93	0.62
Ec. faecium 96	0.42	0.56	0.45	0.40	0.90	0.65
Ec. faecium 97	0.36	0.30	0.63	0.48	1.05	1.04
Ec. durans 98	0.27	0.33	0.05	0.04	0.22	0.23
F. durionis CRL 2054	0.64	0.57	1.45	1.45	1.15	1.20
F. fructosus CRL 2032	0.49	0.53	1.14	0.98	1.49	1.48
F. pseudoficulneus CRL 2033	0.69	0.64	1.14	0.99	1.24	1.19
F. tropaeoli CRL 2039	0.54	0.50	1.08	1.12	1.28	1.27
F. tropaeoli CRL 2035	0.67	0.70	1.23	1.17	1.38	1.42
F. tropaeoli CRL 2036	0.50	0.48	1.26	1.25	1.41	1.45
F. tropaeoli CRL 2038	0.53	0.50	1.23	1.32	1.33	1.47
F. tropaeoli CRL 2037	0.45	0.48	1.18	1.17	1.23	1.27
F. tropaeoli CRL 2034	0.59	0.57	1.28	1.22	1.38	1.37
