# Supplementary material for: Biotransformation of Selenium by Lactic Acid Bacteria: Formation of Seleno-Nanoparticles and Seleno-Amino Acids
Source: Front Bioeng Biotechnol. 2020 Jun 12;8:506. doi: 10.3389/fbioe.2020.00506 (PMC7303280; doi:10.3389/fbioe.2020.00506)
Supplement: Supplementary file 2 [file Image_1.pdf]

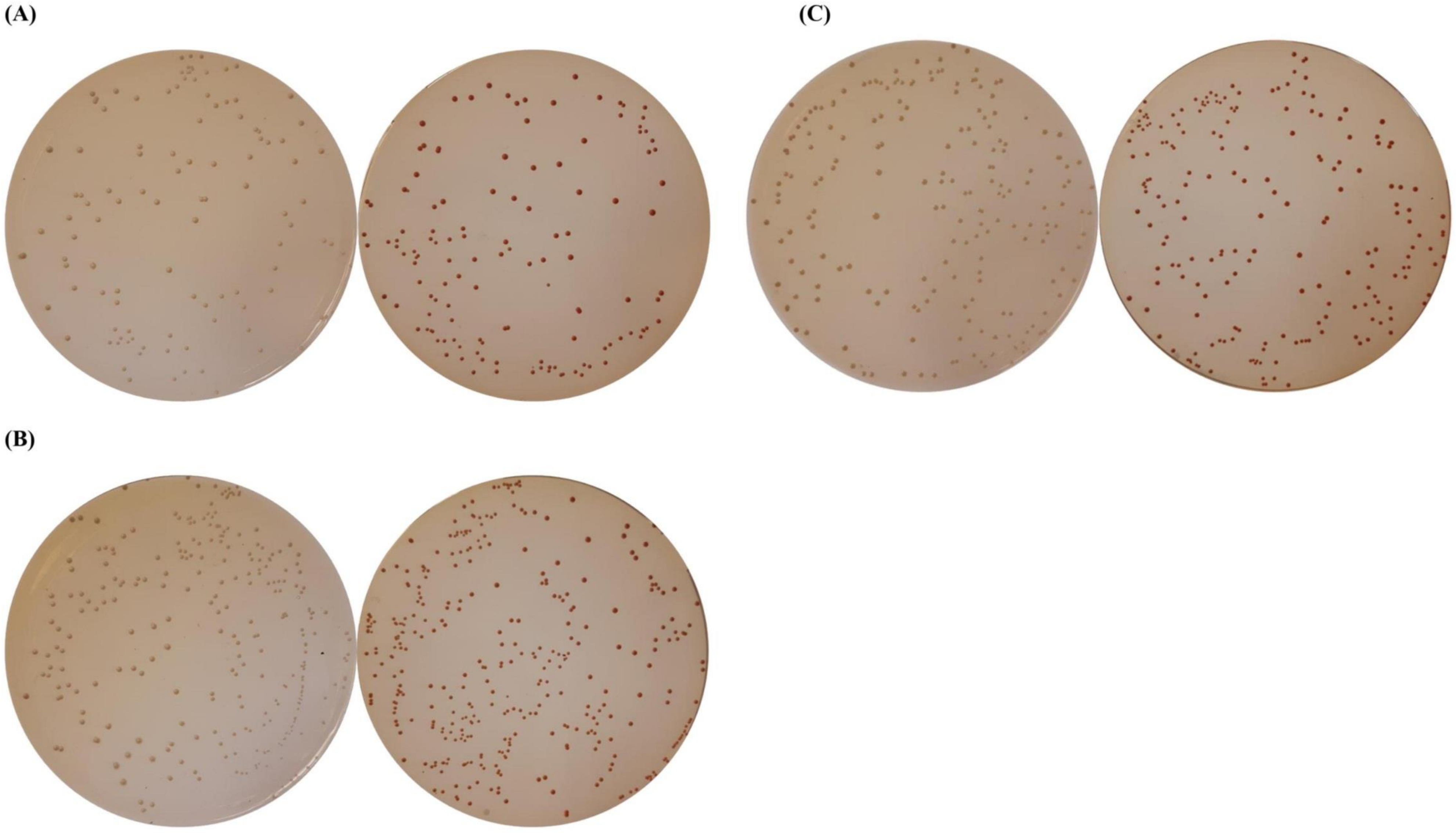

FIGURE S1. Images of colonies of (A) *Lb. brevis* CRL 2051, (B) *Lb. plantarum* CRL 2030, and (C) *F. tropaeoli* CRL 2034 grown in MRS-agar (left) and MRS-Se (right), after incubation at 30 °C during 48 h.
